# Supplementary material for: Imaging the facet surface strain state of supported multi-faceted Pt nanoparticles during reaction
Source: Nat Commun. 2022 May 30;13:3003. doi: 10.1038/s41467-022-30592-1 (PMC9151645; doi:10.1038/s41467-022-30592-1)
Supplement: Supplementary file 1 — Supplementary Information [file 41467_2022_30592_MOESM1_ESM.pdf]

Supplementary Information: Imaging the facet surface strain state of supported multi-faceted Pt nanoparticles during reaction

Maxime Dupraz,[1,2,*] Ni Li,[1,2] Jérôme Carnis,[2,3] Longfei Wu,[2,3] Stéphane Labat,[3] Corentin Chatelier,[1,2] Rim van de Poll,[4] Jan P. Hofmann,[4,5] Ehud Almog,[6] Steven J. Leake,[2] Yves Watier,[2] Sergey Lazarev,[7] Fabian Westermeier,[7] Michael Sprung,[7] Emiel J.M. Hensen,[4] Olivier Thomas,[3] Eugen Rabkin,[6] and Marie-Ingrid Richard[1,2,†]

[1]*Univ. Grenoble Alpes, CEA Grenoble, IRIG, MEM, NRS, 17 rue des Martyrs 38000 Grenoble, France*
[2]*ESRF - The European Synchrotron, 71 Avenue des Martyrs, Grenoble 38000, France*
[3]*Aix Marseille Université, CNRS, Université de Toulon, IM2NP UMR 7334, 13397 Marseille, France*
[4]*Laboratory for Inorganic Materials and Catalysis, Department of Chemical Engineering and Chemistry, Eindhoven University of Technology, P. O. Box 513, 5600 MB Eindhoven, The Netherlands*
[5]*Surface Science Laboratory, Department of Materials and Earth Sciences, Technical University of Darmstadt, Otto-Berndt-Strasse 3, 64287 Darmstadt, Germany*
[6]*Department of Materials Science and Engineering, Technion-Israel Institute of Technology, 3200003, Haifa, Israel*
[7]*Deutsches Elektronen-Synchrotron (DESY), D-22607 Hamburg, Germany*

*Electronic address: `maxime.dupraz@esrf.fr`
†Electronic address: `mrichard@esrf.fr`

S1. SCANNING ELECTRON MICROSCOPY (SEM) IMAGES OF THE SAMPLE

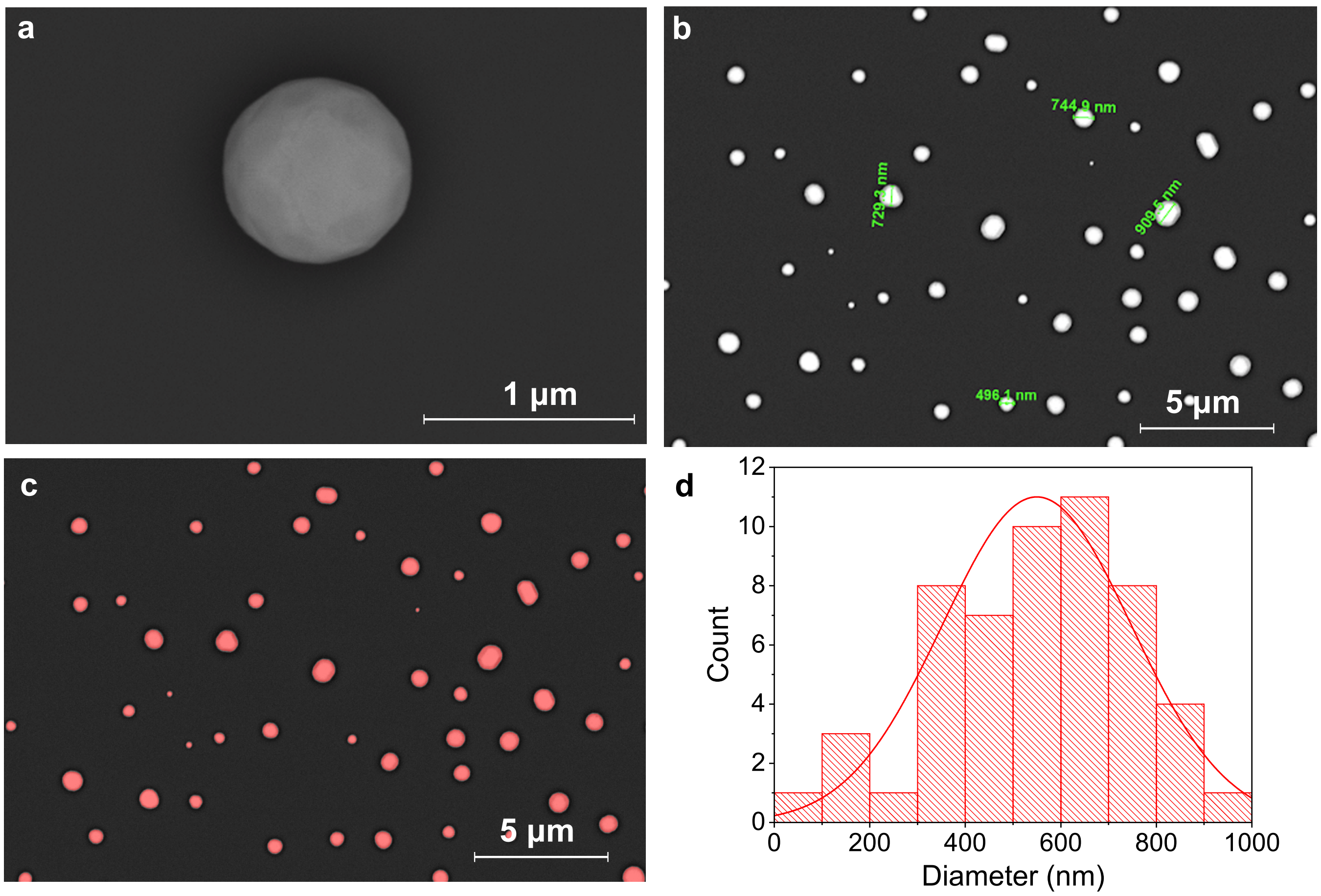

FIG. S1: **SEM images of the Pt samples.** **a** Close up view of a faceted 800-nm Pt NP with a shape similar to NP650. **b** Overview of the [1 1 1]-oriented solid state dewetted Pt NPs. **c** A threshold has been used to detect the particles. **d** Histogram of the diameter of the particles from the thresholded SEM image (c).

S2. OVERVIEW OF THE GAS CYCLES FOR ALL THE MEASURED PARTICLES.

			Gas mixture (%)						Time per cycle (hh:mm)	
Scan number	BCDI scans	T(°C)	Ar	O_2	CO	CO:O ratio	Cycle type	Particle	First scan	Total time
S165	1	25	100	0	0	N.A	N.A	NPD1	0:30	
S172 → S179	2	50	100	0	0	N.A	N.A	NPD1	0:22	0:44
S196	1	150	100	0	0	N.A	N.A	NPD1	0:48	
S202	1	225	100	0	0	N.A	N.A	NPD1	0:29	
S209	1	275	100	0	0	N.A	N.A	NPD1	0:57	
S226 → S234	3	325	100	0	0	N.A	N.A	NPD1	0:49	1:24
S240	1	375	100	0	0	N.A	N.A	NPD1	0:25	
S250 → S265	3	425	100	0	0	N.A	N.A	NPD1	0:26	1:43
S274 → S278	2	450	100	0	0	N.A	N.A	NPD1	0:25	0:41
S280 → S347	7	450	98	2	0	N.A	N.A	NPD1	0:20	2:33
S353 → S421	5	450	94	2	4	2	SC1	NPD1	0:21	3:26
S426 → S457	6	450	92	4	4	1	OC1	NPD1	0:09	1:43
S462 → S482	5	450	98	2	0	N.A	N.A	NPD1	0:14	1:16
S487 → S540	6	450	94	2	4	2	SC2	NPD1	0:10	1:55
S543 → S559	5	450	96	0	4	N.A	N.A	NPD1	0:11	1:00
S597 → S615	5	450	96	0	4	N.A	N.A	NPD2	2:37	3:34
S622 → S634	5	450	100	0	0	N.A	N.A	NPD2	0:11	0:55
S639 → S679	11	450	98	2	0	N.A	N.A	NPD2	0:10	1:48
S684 → S688	2	450	94	2	4	2	SC3	NPD2	0:26	0:35
S689 → S697	2	450	88	4	8	2	SC3'*	NPD2	0:07	0:27
S702 → S710	3	450	96	0	4	N.A	N.A	NPD2	0:10	0:37
S715	1	450	100	0	0	N.A	N.A	NPD2	0:10	
S726	1	450	100	0	0	N.A	N.A	NPD1	1:14	
S787 → S800 (a)	3	450	100	0	0	N.A	N.A	NP650	3:20	4:00
S805 → S824 (b)	4	450	98	2	0	N.A	N.A	NP650	0:12	1:25
S828 → S839 (c)	4	450	97.5	2.5	0	N.A	N.A	NP650	0:15	0:50
S842 → S856 (d)	5	450	72.5	2.5	25	10	RC1	NP650	0:12	1:01
S859 → S864 (e)	2	450	100	0	0	N.A	N.A	NP650	0:15	0:29
S885 → S905 (a)	5	450	100	0	0	N.A	N.A	NP300	1:27	2:46
S910 → S922 (b)	4	450	97.5	2.5	0	N.A	N.A	NP300	0:14	0:59
S926 → S946 (c)	6	450	72.5	2.5	25	10	RC2	NP300	0:15	1:30
S950 → S966 (d)	5	450	100	0	0	N.A	N.A	NP300	0:12	1:15
S970 → S986 (e)	5	450	97.5	2.5	0	N.A		NP300	0:10	1:16
S990 (f)	1	450	72.5	2.5	25	10	RC3	NP300	0:19	
S994 → S1010 (g)	5	450	87.5	12.5	0	N.A	N.A	NP300	0:17	1:10
S1016 → S1034 (h)	5	450	62.5	12.5	25	2	SC4	NP300	0:21	1:53
S1038 → S1059 (i)	6	450	75	0	25	N.A	N.A	NP300	0:17	1:03
S1065 → S1081 (j)	5	450	100	0	0	N.A	N.A	NP300	0:14	0:50
S1085 → S1100 (k)	5	450	87.5	12.5	0	N.A	N.A	NP300	0:13	0:48
S1105 → S1130	9	450	62.5	12.5	25	2	SC5	NP300	0:03	0:48
S1131 → S1159	9	450	75	0	25	N.A	N.A	NP300	0:00	1:10
S1167 → S1171	2	450	87.5	12.5	0	N.A	N.A	NP300	0:17	0:27
S1175 → S1179	2	450	87.5	12.5	0	N.A	N.A	NP650	0:46	0:59
S1183 → S1199	5	450	62.5	12.5	25	2	SC6	NP650	0:14	1:02
S1203 → S1244	5	450	75	0	25	N.A	N.A	NP650	0:13	1:48

TABLE S1: Overview of the gas cycles carried out on all the measured nanoparticles, the time spent in each cycle as well as the number of BCDI scans measured for a given gas condition and the temperature at which the measurement was carried out. Two measurements carried out on NPD1 are shown in Supplementary Figure S2. N.A. stands for not applicable, while OC, SC and RC mean oxidizing, stoichiometric and reducing conditions, respectively. The time specified in the column "First scan" refers to the time elapsed between a change in temperature and/or gas condition and the actual measurement of the first BCDI scan. *SC3 and SC3' are considered to belong to the same cycle. Indeed, they are performed sequentially with the same ratio between reactants ($\chi = 2$), even though the fraction of CO and O_2 in the gas mixture is doubled during SC3'.

The BCDI scans shown in this work were collected during two CO oxidation cycles in reducing conditions (RC2 and RC3) and one cycle in stoichiometric conditions (SC4) for NP300; one in reducing conditions (RC1) and one in stoichiometric conditions (SC6) for NP650. Since all the particles were measured on the same substrate, NP650 also experienced 3 stoichiometric CO oxidation (SC1, SC2 and SC3-SC3', $\chi = 2$) cycles before the first Ar measurement and one oxidizing cycle (OC1, $\chi = 1$), while NP300 underwent 3 stoichiometric CO oxidation (SC1, SC2 and SC3-SC3'), 1 oxidizing (OC1) and 1 reducing (RC1) CO cycle before being measured ($\chi = 10$). The first measurement in Ar was preceded by a 37 minutes reduction of the catalyst at 450°C in 4% CO for NP650 (see S702 → S710 in Table S1), and a CO oxidation in CO excess for NP300 (see RC1 in Table S1), implying that the surface of the NPs was most likely free of O_2 adsorbates in the initial state for both NPs. Finally, 19 hours separate the two cycles shown for NP650. During this time interval, all the measurements were carried out on NP300: 2 reducing (RC2 and RC3) and 2 stoichiometric cycles (SC4 and SC5).

		Gas mixture (%)						Time per cycle (hh:mm)	
Scan number	BCDI scans	Ar	O_2	CO	CO:O ratio	Cycle type	Particle	First scan	Total time
S787 → S800 (a)	3	100	0	0	N.A	N.A	NP650	3:20	4:00
S805 → S824 (b)	4	98	2	0	N.A	N.A	NP650	0:12	1:25
S828 → S839 (c)	4	97.5	2.5	0	N.A	N.A	NP650	0:15	0:50
S842 → S856 (d)	5	72.5	2.5	25	10	RC1	NP650	0:12	1:01
S859 → S864 (e)	2	100	0	0	N.A	N.A	NP650	0:15	0:29
S885 → S905 (a)	5	100	0	0	N.A	N.A	NP300	1:27	2:46
S910 → S922 (b)	4	97.5	2.5	0	N.A	N.A	NP300	0:14	0:59
S926 → S946 (c)	6	72.5	2.5	25	10	RC2	NP300	0:15	1:30
S950 → S966 (d)	5	100	0	0	N.A	N.A	NP300	0:12	1:15
S970 → S986 (e)	5	97.5	2.5	0	N.A		NP300	0:10	1:16
S990 (f)	1	72.5	2.5	25	10	RC3	NP300	0:19	
S994 → S1010 (g)	5	87.5	12.5	0	N.A	N.A	NP300	0:17	1:10
S1016 → S1034 (h)	5	62.5	12.5	25	2	SC4	NP300	0:21	1:53
S1038 → S1059 (i)	6	75	0	25	N.A	N.A	NP300	0:17	1:03
S1065 → S1081 (j)	5	100	0	0	N.A	N.A	NP300	0:14	0:50
S1085 → S1100 (k)	5	87.5	12.5	0	N.A	N.A	NP300	0:13	0:48
S1105 → S1130	9	62.5	12.5	25	2	SC5	NP300	0:03	0:48
S1131 → S1159	9	75	0	25	N.A	N.A	NP300	0:00	1:10
S1167 → S1171	2	87.5	12.5	0	N.A	N.A	NP300	0:17	0:27
S1175 → S1179 (f)	2	87.5	12.5	0	N.A	N.A	NP650	0:46	0:59
S1183 → S1199 (g)	5	62.5	12.5	25	2	SC6	NP650	0:14	1:02
S1203 → S1244 (h)	5	75	0	5	N.A	N.A	NP650	0:13	1:48

TABLE S2: Simplified table giving an overview of the scans shown in the manuscript. N.A. stands for not applicable, while OC, SC and RC mean oxidizing, stoichiometric and reducing conditions, respectively. The time specified in the column "First scan" refers to the time elapsed between a change in temperature and/or gas condition and the actual measurement of the first BCDI scan.

Table S2 is a simplified table showing an overview of the scans and cycles performed on the NP650 and NP300 nanoparticles described in the manuscript. As seen from the table, several scans were generally performed for each gas condition, except for S990.

S3. 3D SURFACE STRAIN FIELD EVOLUTION ϵ_{111} DURING REACTION IN NPD1.

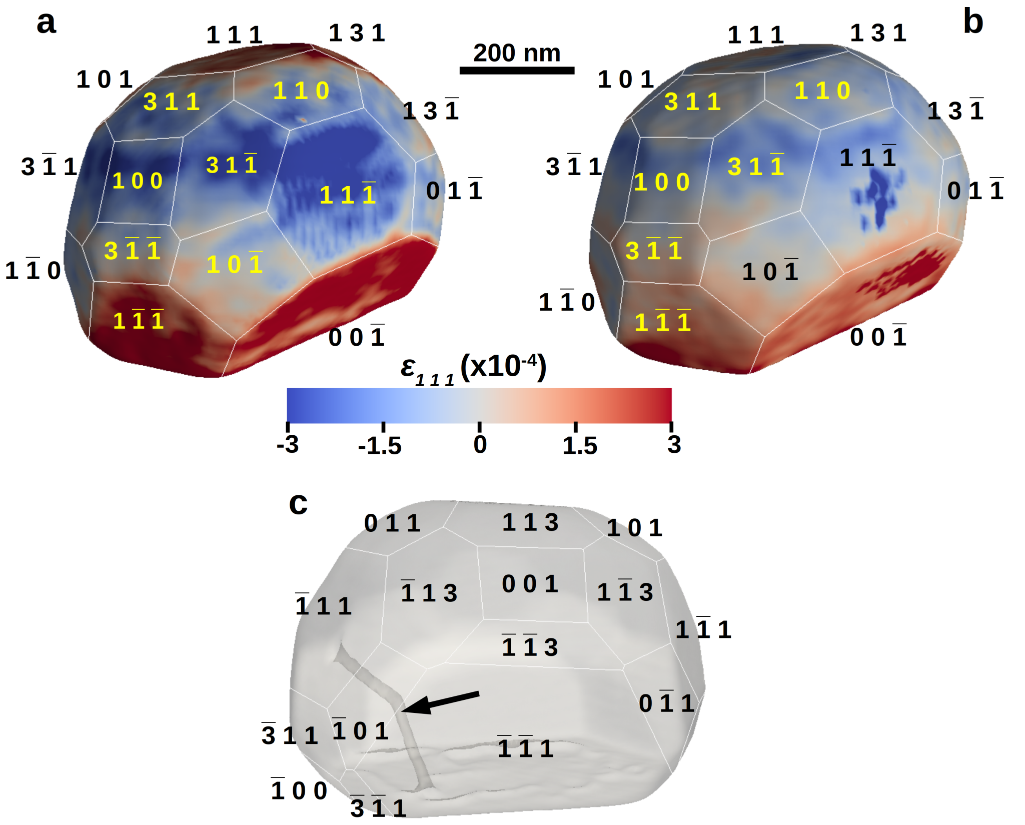

FIG. S2: **Illustration of the strain relaxation in NPD1, a 650 nm NP containing a mixed-type dislocation, during O_2 exposure.** ϵ_{111} strain field in **a** Ar and **b** 2% O_2 and Ar atmosphere. **c** Isosurface drawn at 61% of the maximum of the electron density showing the mixed dislocation (indicated by a black arrow).

Facet	Ar ϵ_{111} (x10^{-4})	Ar + 2% O_2 ϵ_{111} (x10^{-4})
$(1\ \bar{1}\ \bar{1})$	2.96	1.65
$(1\ 1\ \bar{1})$	-2.8	-1.4
$(1\ 0\ 0)$	-2.73	-1.65
$(0\ 0\ \bar{1})$	3.03	1.47
$(1\ 1\ 0)$	-1.13	-1.26
$(1\ \bar{1}\ 0)$	0.18	0.26
$(0\ 1\ \bar{1})$	-1.02	-0.85
$(3\ 1\ 1)$	-1.51	-1.22
$(1\ 3\ \bar{1})$	-0.52	-0.84
$(3\ 1\ \bar{1})$	-3.31	-1.56
$(3\ \bar{1}\ 1)$	-2.77	-1.72
$(3\ \bar{1}\ \bar{1})$	-0.25	0.7

TABLE S3: Average strain (ϵ_{111}) evolution per facet in NPD1 during O_2 exposure for the facets shown in Supplementary Fig. S2.

Figures S2 and Table S3 show the strain evolution in 2% O_2 and Ar atmosphere for a third NP, NPD1, which

was measured before NP300 and NP650. Note that the corresponding BCDI scans (S278 for Ar and S339 for Ar + 2% O_2) were collected at the beginning of the first stoichiometric cycle and correspond to the first exposure of the sample to a gas mixture containing oxygen (see Table S1). Unlike NP300 and NP650, this NP is defective and contains a mixed-type dislocation (Fig. S2c). Despite the presence of this defect and the initial "pristine state" of the NP, the strain evolution during O_2 exposure is relatively consistent with the trends observed in the defect free NPs. In particular, the $\{1\ 0\ 0\}$ and $\{1\ \bar{1}\ 1\}$ and to some extent the $\{1\ \bar{1}\ \bar{3}\}$ facets experience a significant relaxation. In addition, the initial surface strain is also quite similar to the one observed in NP650 and NP300. The differences in the initial strain distribution can be attributed to the different shape of the NP (large truncated $(0\ 0\ \bar{1})$ facet for instance) and to the presence of a mixed dislocation.

S4. OVERVIEW OF THE SCANS MEASURED DURING THE HEATING RAMP

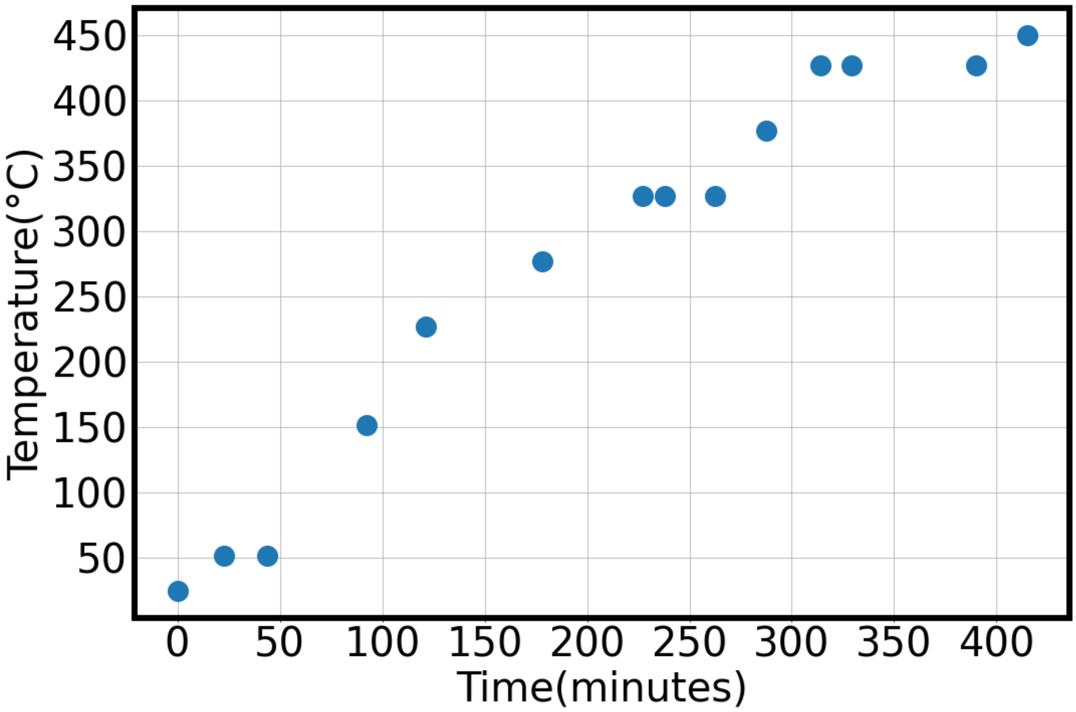

FIG. S3: **Overview of the scans measured during the heating ramp in Ar atmosphere.**

Fig. S3 gives an overview of the scans measured during the heating ramp carried out in Ar atmosphere. The heating ramp was set to 25°C/min, several data points were measured during the heating of the sample. Therefore, it took approximately 415 minutes to reach the reaction temperature of 450°C.

S5. COHERENT DIFFRACTION PATTERNS

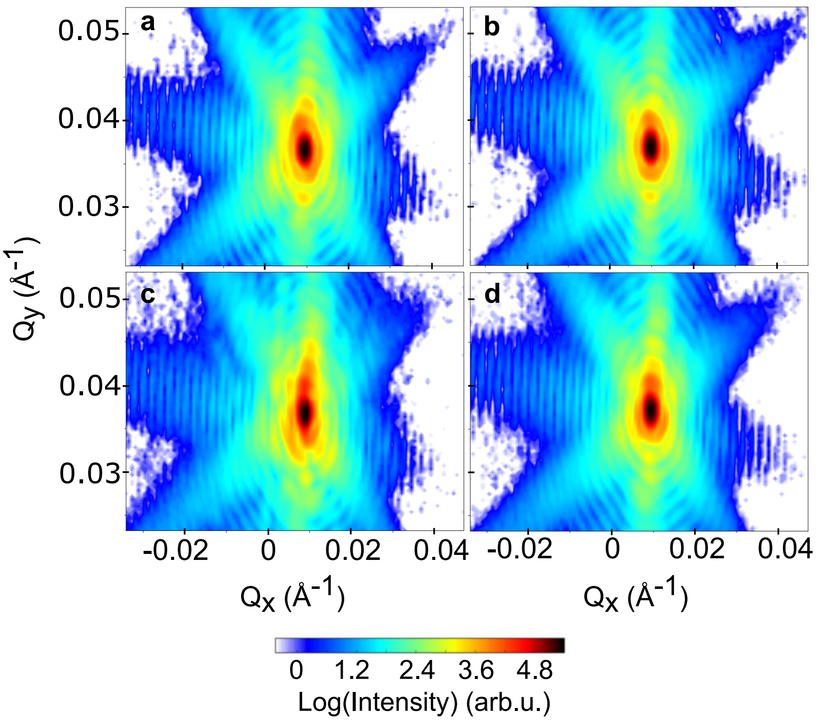

FIG. S4: ***In situ* coherent x-ray diffraction measurements.** **111** Pt Bragg peak of a 300-nm diameter Pt particle at 450°C (NP300), as a function of the in-plane coordinates of the scattering vector **q** (q_x and q_y, q_x being along the x-ray beam) and for different gas flows: **a** Ar, **b** 2.5% O_2 and Ar, **c** CO oxidation: 12.5% O_2, 25% CO and Ar and **d** 12.5% O_2 and Ar.

Figure S4 displays coherent x-ray diffraction measurements as a function of the in-plane coordinates of the scattering vector **q** (q_x and q_y; q_x,q_y and q_z being along the incident x-ray beam (downstream), outboard and vertically upward, respectively) for different gas compositions for NP300: a) Ar, b) 2.5% O_2 and Ar, c) 12.5% O_2, 25% CO and Ar and d) 12.5% O_2 and Ar. The third gas flow corresponds to CO oxidation in stoichiometric conditions ($CO + \frac{1}{2}\ O_2 \rightarrow CO_2$). All the diffraction patterns display well defined streaks indicating the faceted nature of the NP surface. The diffraction patterns are all asymmetric along the q_x direction, reflecting the asymmetry of the local strain distribution inside the investigated particle. Interestingly, large changes are observed in the vicinity of the **111** Bragg peak during CO oxidation (see the first fringes in Fig. S4c). These changes arise from local structural variations and suggest that the structure of the Pt particle strongly evolves during CO oxidation.

S6. SPATIAL RESOLUTION

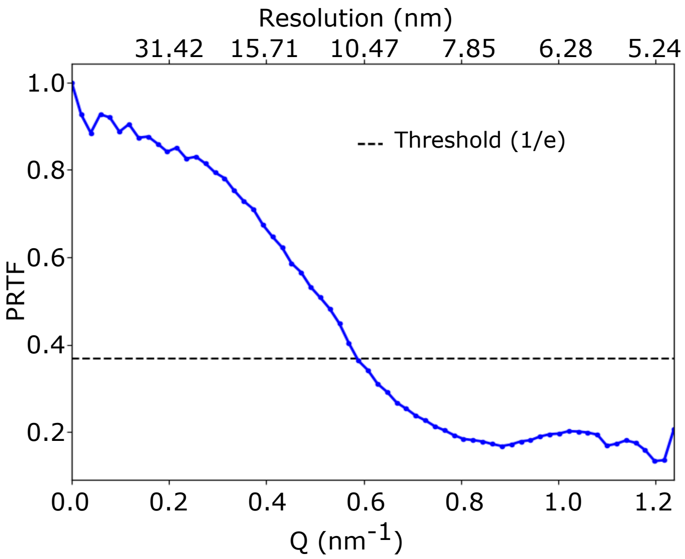

FIG. S5: **Phase-Retrieval Transfer Function (PRTF) calculated from one measurement**. A threshold of 1/e is used to estimate the spatial resolution.

Several methods can be used to estimate the spatial resolution. The resolution of the reconstruction was first estimated using the Phase-Retrieval Transfer Function (PRTF) [1, 2]. PRTF in Bragg coherent x-ray imaging is defined as:

$$PRTF(q_i) = \frac{\sum_{q \in q_i} \mid \tilde{O}(q) \mid}{\sum_{q \in q_i} \sqrt{I(q)}} \tag{1}$$

where q is the scattering vector magnitude and corresponds to the radial distance from the center of the diffraction pattern. The summations are over all q voxels that are contained in the shell q_i. $\tilde{O}(q)$ is the inverse Fourier Transform of the reconstructed object and $I(q)$ is the measured intensity. PRTF reveals the similarity between the amplitude of the retrieved pattern and the measured pattern with respect to q. This leads to a spatial resolution of 10.5 nm.

S7. FACET SUBFAMILIES

Subfamily	Facets	α (°)	NP300	NP650
1 1 1	(111), $(\bar{1}\bar{1}\bar{1})$	0, 180	2	2
1 $\bar{1}$ 1	$(1\bar{1}1)$, $(11\bar{1})$, $(\bar{1}11)$, $(1\bar{1}\bar{1})$, $(\bar{1}\bar{1}1)$, $(\bar{1}1\bar{1})$	70.5, 180-70.5	6	6
1 0 0	(100), (010), (001), $(\bar{1}00)$, $(0\bar{1}0)$, $(00\bar{1})$	54.7, 180-54.7	6	6
1 1 0	(110), (011), (101)	35.3	3	3
1 $\bar{1}$ 0	$(1\bar{1}0)$, $(0\bar{1}1)$, $(\bar{1}01)$, $(\bar{1}10)$, $(01\bar{1})$, $(10\bar{1})$	90	6	6
1 1 $\bar{3}$	$(\bar{1}3\bar{1})$, $(3\bar{1}\bar{1})$, $(\bar{1}\bar{1}3)$, $(1\bar{3}1)$, $(\bar{3}11)$, $(11\bar{3})$	80, 180-80	4	5
1 $\bar{1}$ $\bar{3}$	$(13\bar{1})$, $(31\bar{1})$, $(3\bar{1}1)$, $(1\bar{1}3)$, $(\bar{1}13)$, $(\bar{1}31)$<br>$(\bar{1}\bar{3}1)$, $(\bar{3}\bar{1}1)$, $(\bar{3}1\bar{1})$, $(\bar{1}1\bar{3})$, $(1\bar{1}\bar{3})$, $(1\bar{3}\bar{1})$	58.5, 180-58.5	5	12
1 1 3	(113), (131), (311)	29.5	0	3
Total			32	43

TABLE S4: Overview of the facet subfamilies for the two NPs.

S8. SLICES OF THE RECONSTRUCTED ELECTRON DENSITY AND AMPLITUDE HISTOGRAM FOR NP650.

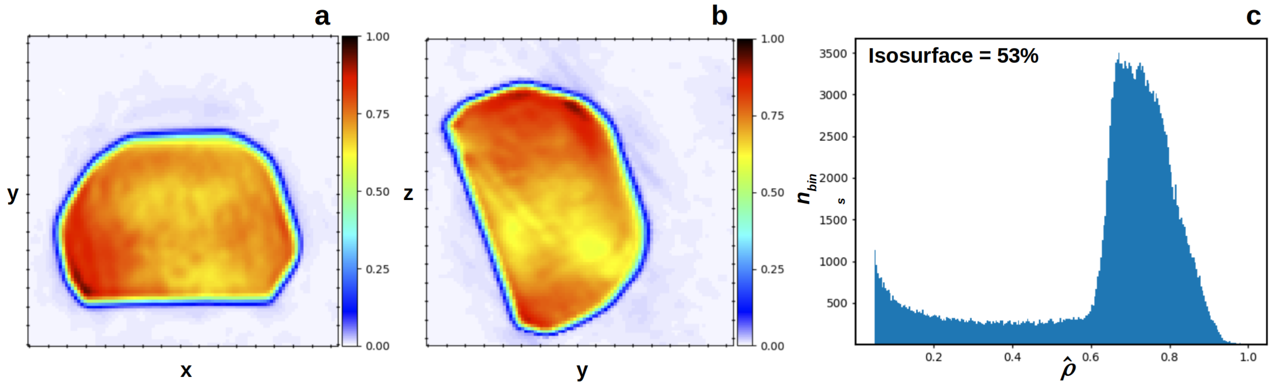

FIG. S6: **Fluctuations in the reconstructed Bragg electron density. a-b** Slices of the reconstructed electron density for NP650 in its initial state in Ar atmosphere (S787). **c** Histogram of the reconstructed electron density, based on the criterion described in Ref. [3]. The voxels belonging to the NP have roughly a Gaussian distribution centered around 0.72 with a standard deviation of 0.19. This value of 53% corresponds to the average of this Gaussian distribution subtracted by its standard deviation, and it is a good estimate for the choice of the isosurface on the surface strain distribution.

S9. 3D SURFACE DISPLACEMENT FIELD EVOLUTION u_{111} DURING REACTION IN NP650.

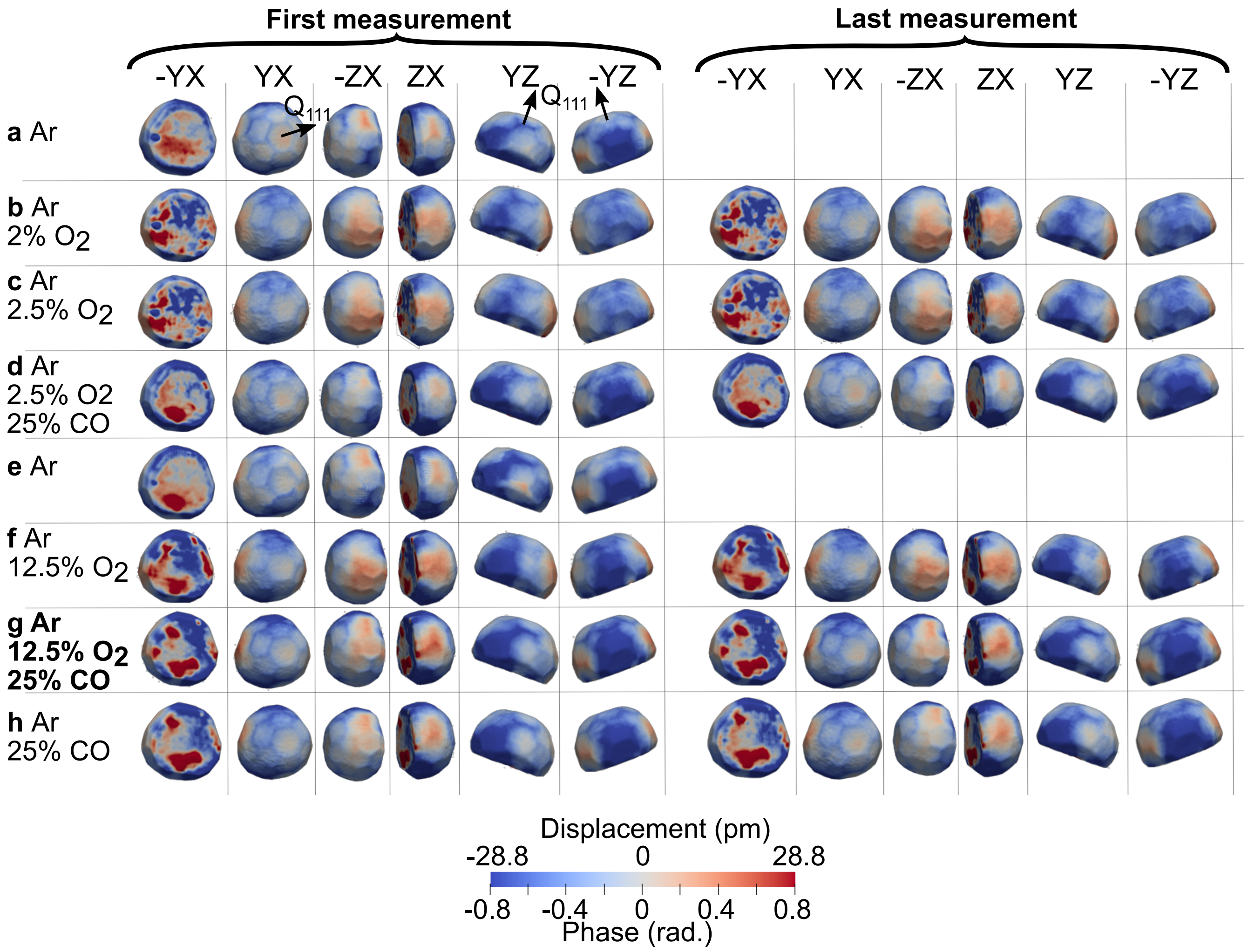

FIG. S7: ***In situ* 3D displacement field images during reaction in NP650.** Evolution of BCDI reconstructions of the displacement field along the [111] direction, drawn at 50% of the maximum Bragg electron density of a 650-nm diameter Pt nanoparticle (NP650) at 450°C and for different gas mixtures (CO oxidation reaction under stoichiometric conditions is indicated in bold): **a** Ar, **b** Ar + 2% O_2, **c** Ar + 2.5% O_2, **d** Ar, 2.5% O_2 + 25% CO, **e** Ar, **f** Ar + 12.5% O_2, **g** Ar, 12.5% O_2 + 25% CO and **h** Ar + 25% CO. The left (right) panel corresponds to the first (last) measurement of the corresponding gas mixture. During condition **a** and **e** only a single measurement was taken and is thus displayed. The direction of the scattering vector $\mathbf{q}_{111}$ from several fields of view is indicated for some images of the top row (condition **a**).

S10. 3D SURFACE STRAIN FIELD EVOLUTION ϵ_{111} DURING REACTION IN NP300.

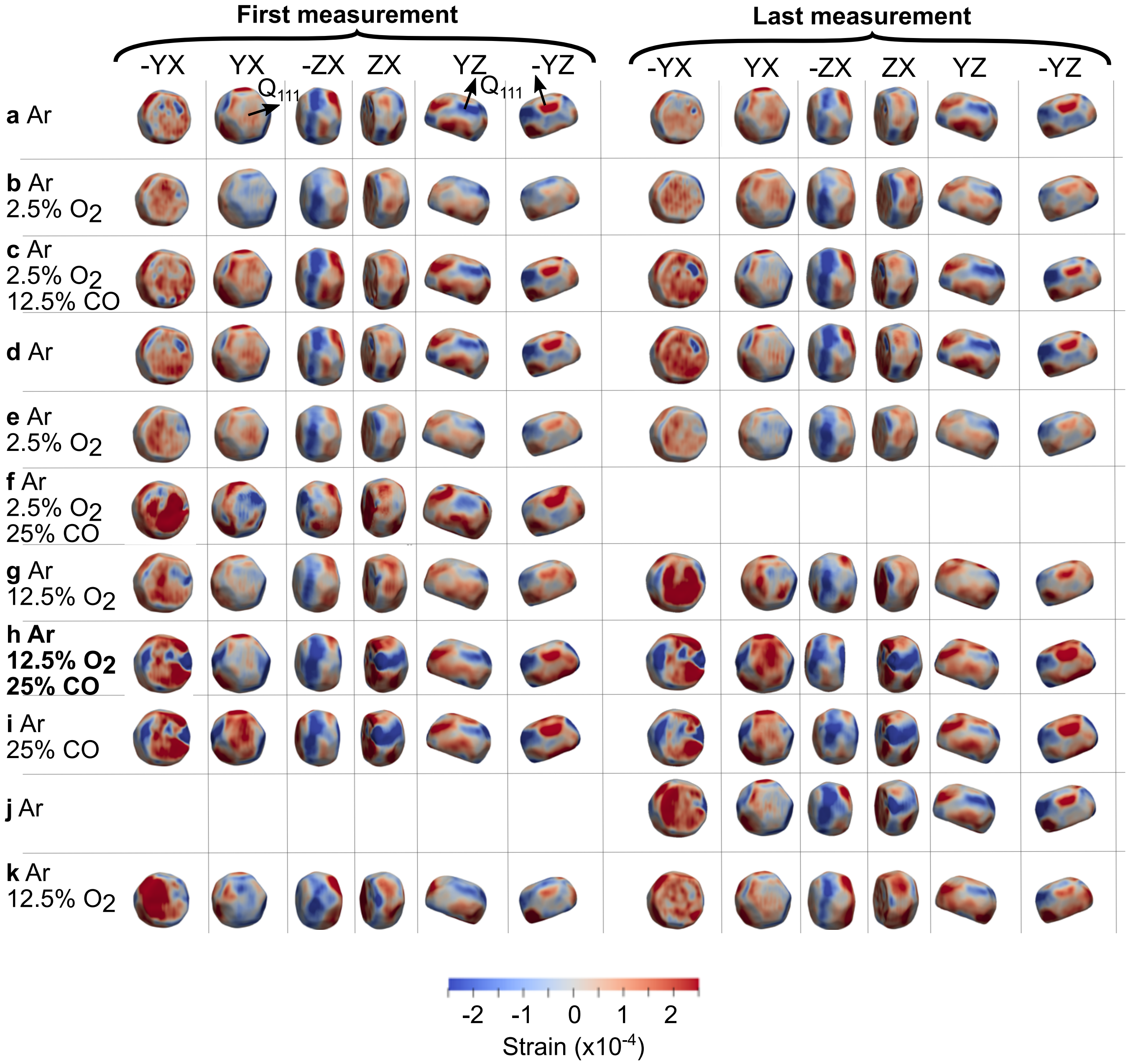

FIG. S8: ***In situ* 3D strain field evolution during reaction in NP300.** Evolution of BCDI reconstructions of the ϵ_{111} strain field, drawn at 50% of the maximum Bragg electron density of the 300-nm diameter Pt nanoparticle (NP300) at 450°C and for different gas mixtures (CO oxidation reaction under stoichiometric conditions is indicated in bold): **a** Ar, **b** Ar + 2.5% O_2, **c** Ar, 2.5% O_2 + 12.5% CO, **d** Ar, **e** Ar + 2.5% O_2, **f** Ar + 2.5% O_2 + 25% CO, **g** Ar + 12.5% O_2, **h** Ar + 12.5% O_2 + 25% CO, **i** Ar + 25% CO, **j** Ar and **k** Ar + 12.5% O_2. The left (right) panel corresponds to the first (last) measurement of the corresponding gas mixture.The direction of the scattering vector $\mathbf{q}_{111}$ from several fields of view is indicated for some images of the top row (condition a).

S11. 3D SURFACE STRAIN FIELD EVOLUTION ϵ_{111} DURING REACTION IN NP650.

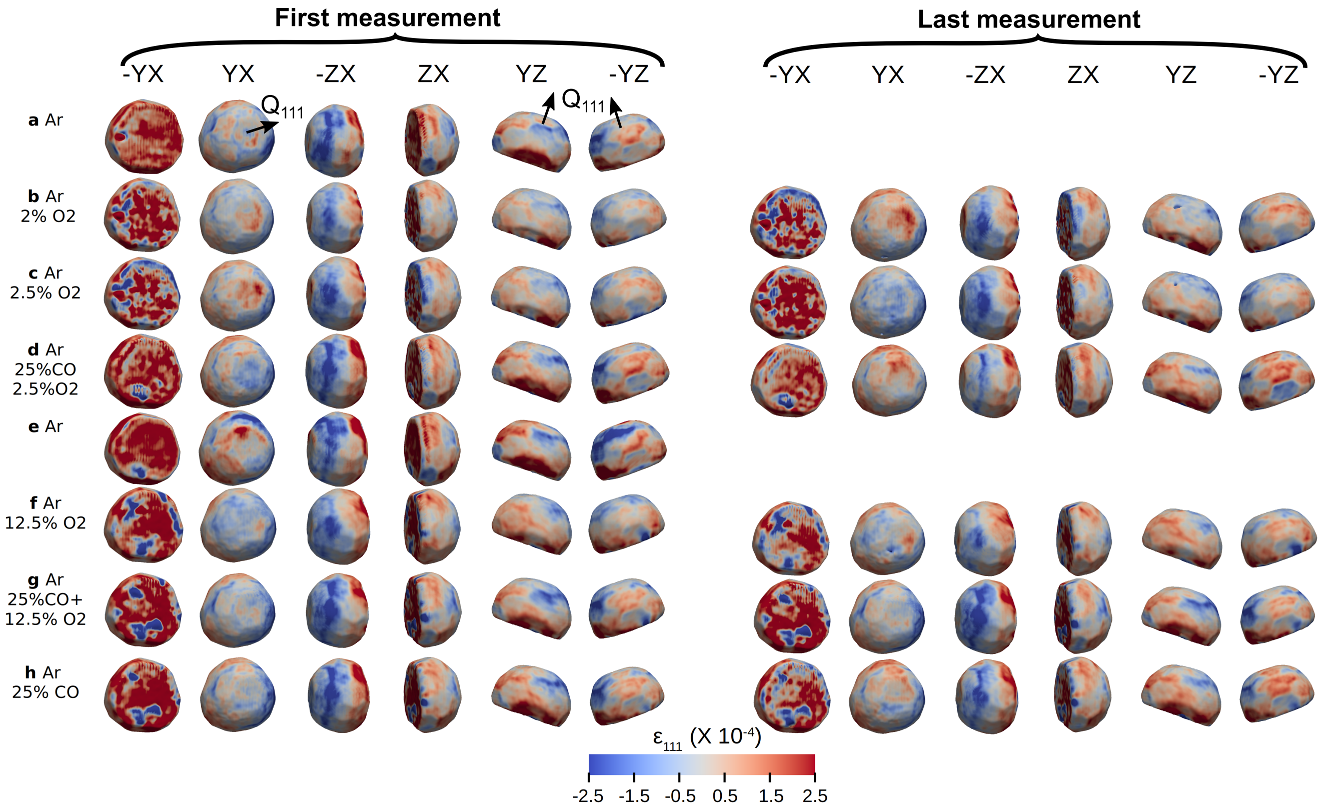

FIG. S9: ***In situ* 3D strain field images during reaction in NP650.** Evolution of BCDI reconstructions of the strain field along the [111] direction, drawn at 50% (see Figure S6 for the choice of this value for the isosurface) of the maximum Bragg electron density of a 650-nm diameter Pt nanoparticle (NP650) at 450°C and for different gas mixtures: **a** Ar, **b** Ar + 2% O_2, **c** Ar + 2.5% O_2, **d** Ar, 2.5% O_2 + 25% CO, **e** Ar, **f** Ar + 12.5% O_2, **g** Ar, 12.5% O_2 + 25% CO and **h** Ar + 25% CO. The left (right) panel corresponds to the first (last) measurement of the corresponding gas mixture. During condition **a** and **e** only a single measurement was taken and is thus displayed. The direction of the scattering vector $\mathbf{q}_{111}$ from several fields of view is indicated for some images of the top row (condition **a**).

S12. SENSITIVITY OF BCDI TO LATTICE DISPLACEMENT AND STRAIN

BCDI allows to reconstruct a 3D complex image of isolated objects such as NPs. Its amplitude corresponds to the electron density of the object, while its phase corresponds to a projection of the 3D displacement field $\mathbf{u}(\mathbf{r})$ onto the scattering vector (111, in the present work). In the kinematic approximation of scattering, which is fully justified here because we study small crystals (<1μm), the scattered intensity is the square modulus of the Fourier transform of the atomic scattering factor:

$$I(\mathbf{q}) = |\int f(\mathbf{r}) e^{2i\pi \mathbf{q}.\mathbf{r}}|^2 \tag{2}$$

where the integration is performed over the illuminated volume, f($\mathbf{r}$), $\mathbf{q}$ and $\mathbf{r}$ being the atomic scattering factor, the scattering vector and the positions of the atoms, respectively. The Bragg geometry probes the crystalline order and coherent X-rays can be used in Bragg geometry to investigate the deviation of the sample from a perfect crystal order.

For an imperfect crystal, one can define $\mathbf{r}_0$ the positions of a perfect lattice that approximates the crystal and $\mathbf{u}(\mathbf{r})$ the displacement of the atoms from the perfect lattice such that $\mathbf{r} = \mathbf{r}_0 + \mathbf{u}(\mathbf{r})$ (see Fig. S10). Now let's consider a Bragg reflection with a reciprocal space vector $\mathbf{g}$ (defined on the perfect lattice that approximates the crystal). We focus on a region of the reciprocal space (RS) in the vicinity of $\mathbf{g}$: the phase factor defined in the exponential of Eq.2 can be decomposed as follows:

$$\mathbf{q}.\mathbf{r} = \mathbf{q}.\mathbf{r}_0 + \mathbf{g}.\mathbf{u}(\mathbf{r}) + (\mathbf{q} - \mathbf{g}).\mathbf{u}(\mathbf{r}) \tag{3}$$

The third term in Eq. 3 can be neglected if ($\mathbf{q}$ - $\mathbf{g}$).$\mathbf{u}(\mathbf{r}) << 1$ (Takagi's approximation), which is equivalent to assuming small distortions of the lattice and a restricted extent of the RS, two perfectly reasonable assumptions in the present work. This gives:

$$I(\mathbf{q}) = |FT[\tilde{f}(\mathbf{r})]|^2 \text{ with } \tilde{f}(\mathbf{r}) = f(\mathbf{r}) e^{2i\pi \mathbf{g}.\mathbf{u}(\mathbf{r})} \tag{4}$$

Here, we consider only non-resonant scattering, such that the atomic scattering factor f($\mathbf{r}$) is essentially the electron density of the sample $\rho(\mathbf{r})$, while the modified scattering factor $\tilde{f}(\mathbf{r})$ is referred as the complex electron density $\tilde{\rho}(\mathbf{r})$: its modulus is the physical electron density while its phase encodes the projection of the displacement field onto the diffraction vector $\mathbf{g}$.

This displacement field $\mathbf{u}(\mathbf{r})$ contained in the phase term: $\phi = \mathbf{g}.\mathbf{u}(\mathbf{r})$ can be understood by considering a block of material which is displaced from the rest of the lattice by a vector $\mathbf{u}(\mathbf{r})$ as illustrated in Fig. S10. The phase of the x-ray wave scattered by this block of atoms is shifted relative to the rest of the reference crystal by an amount $\phi = \mathbf{g}.\mathbf{u}(\mathbf{r})$ provided that a complex image of the sample is obtained, the phase shift appears in the reconstructed image as a region of complex density with the same amplitude but a different phase:

The strain is then simply a derivative of the reconstructed displacement. For more information, please refer to Refs. [4, 5].

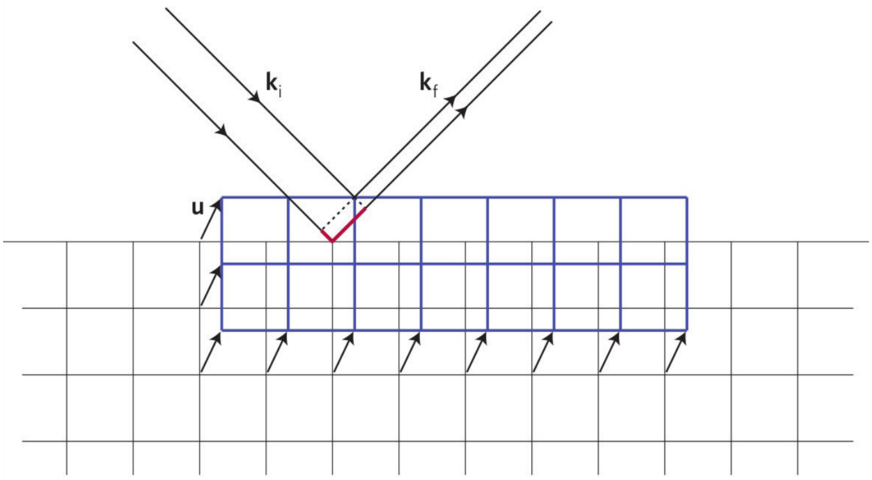

FIG. S10: **Sensitivity of coherent X-rays to lattice displacements** (image taken from Ref. [4])

S13. MACROSCOPIC STRAIN AND STRAIN FIELD ENERGY OF NP650.

Note that the strain field energy (see Supplementary Fig. S11(c)) is higher for NP650 compared to NP300. This is maybe due to the larger size of the particle.

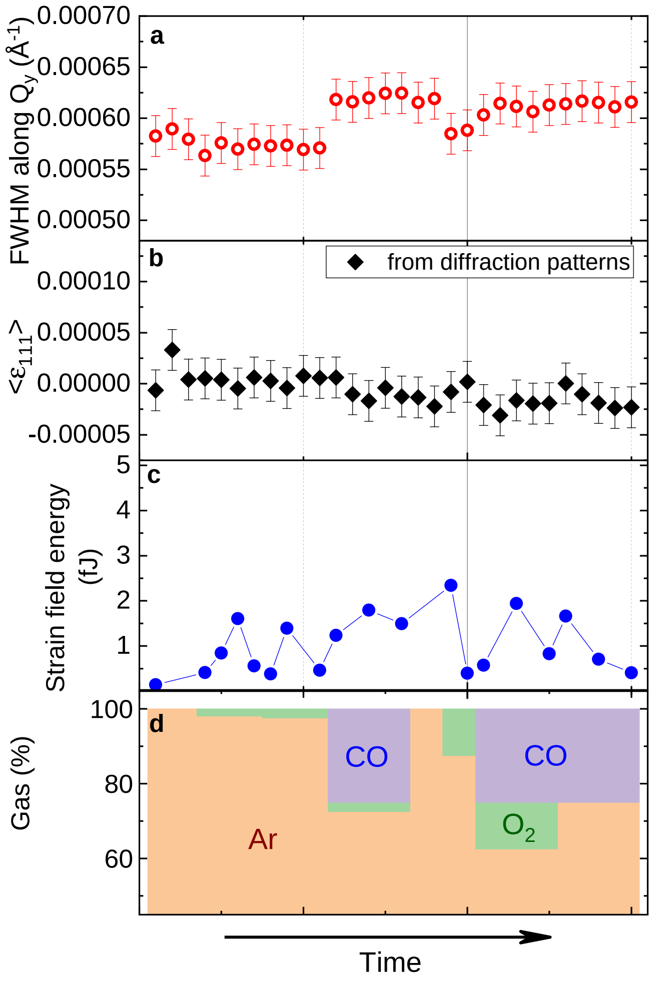

FIG. S11: ***In situ* structural evolution of NP650 during reaction.** Evolution of **a** the full width at half maximum (FWHM) of its diffraction pattern along the Q_y direction, of **b** its average strain, $\langle \epsilon_{111} \rangle$ calculated from the center of mass of the diffraction patterns, of **c** its strain field energy and of **d** the gas composition at the inlet (Ar, O_2 and CO are in orange, green and purple, respectively) as a function of time. The dashed line indicates the beginning of the CO oxidation reaction under stoichiometric conditions. The error bars represent the standard deviation of the measured values.

S14. COMPARISON WITH THE 3D STRAIN FIELD OBTAINED FROM MS CALCULATIONS FOR NP650

Family	Exp. NP300 ϵ_{111} (x10^{-4})	Exp. NP650 ϵ_{111} (x10^{-4})	Simu. NP300 ϵ_{111} (x10^{-4})	Simu. NP650 ϵ_{111} (x10^{-4})
1 1 1	0.76	0.07	0.76	0.52
1 $\bar{1}$ 1	-0.75	-0.5	-0.43	-0.42
1 0 0	0.78	0.66	0.22	0.49
1 1 0	0.56	-0.09	0.32	0.49
1 $\bar{1}$ 0	0.19	0.38	0.09	0.21
1 1 $\bar{3}$	1.75	1.40	2.21	1.32
1 $\bar{1}$ $\bar{3}$	0.96	0.55	0.02	0.15
1 1 3	N.A	-0.41	N.A	0.29

TABLE S5: Initial average strain (ϵ_{111}) per facet in Ar atmosphere for the different facet subfamilies in the two NPs. Comparison with MS calculations: the simulated NPs have the same size (200 nm) and voxel size (6.7 nm) corresponding to the experimental voxel size and their faceting corresponds to NP300 and NP650.

S15. COMPARISON BETWEEN EXPERIMENT AND SIMULATION FOR NP300.

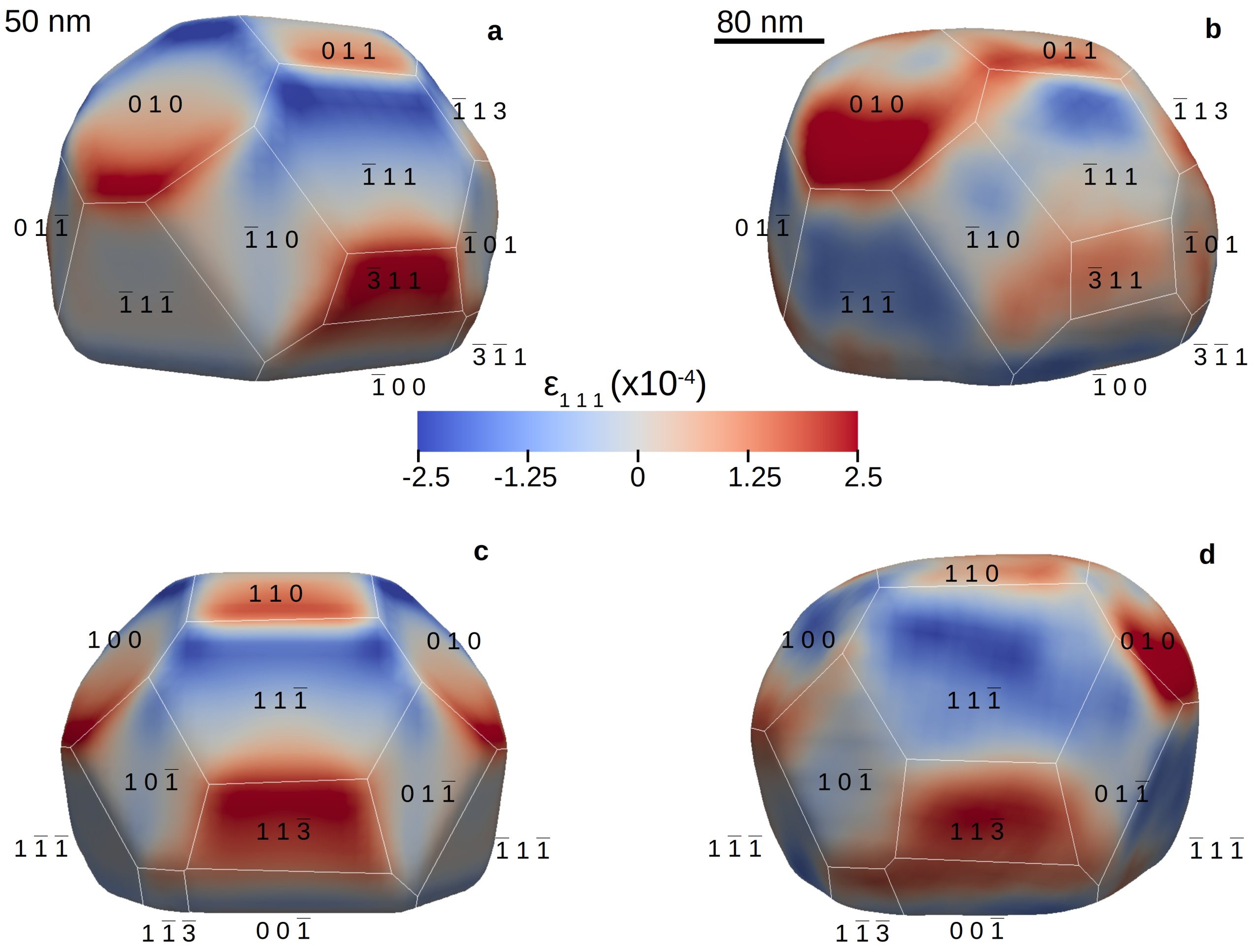

FIG. S12: **Comparison between experiment and simulation for NP300**. **a**, **c** 200 nm simulated nanoparticle with the same faceting as NP300 and relaxed by energy minimization seen from two different field of views. **b**, **d** Experimental ϵ_{111} strain field measured in Ar atmosphere for NP300 and seen from the same field of views.

S16. DISPLACEMENT u_{111} AND STRAIN ϵ_{111} FIELDS EVOLUTION FOR DIFFERENT GAS CONDITIONS IN NP300.

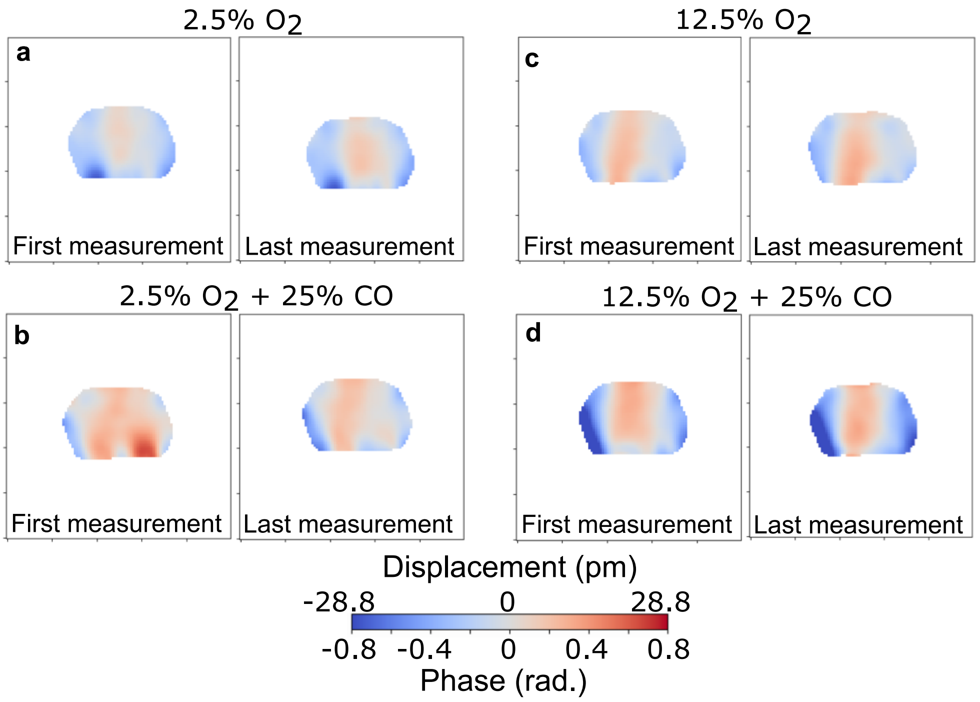

FIG. S13: **Slice of the displacement field.** Slice through the center of NP300 showing the evolution of the displacement field u_{111} for the first and last measurements of several gas conditions: **a** 2.5% O_2, **b** 12.5% O_2, **c** 2.5% O_2 + 25% CO and **d** 12.5% O_2 + 25% CO.

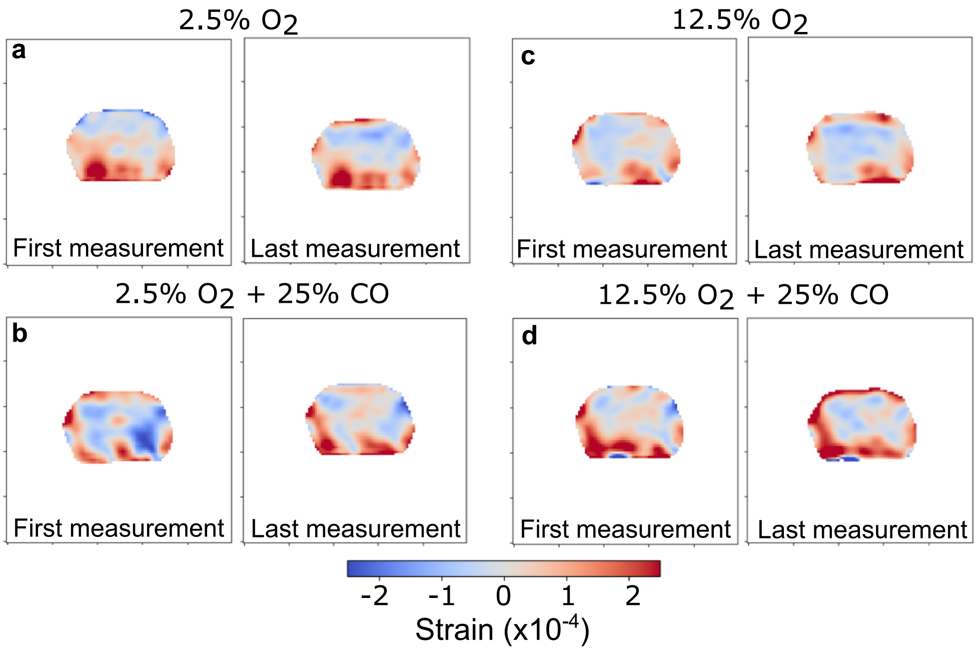

FIG. S14: **Slice of the strain field.** Slice through the center of NP300 showing the evolution of the strain field ϵ_{111} for the first and last measurements of several gas conditions: **a** 2.5% O_2, **b** 12.5% O_2, **c** 2.5% O_2 + 25% CO and **d** 12.5% O_2 + 25% CO.

S17. AVERAGE SURFACE DISPLACEMENT AND STRAIN COMPUTED USING THE FACET ANALYSER PLUGIN.

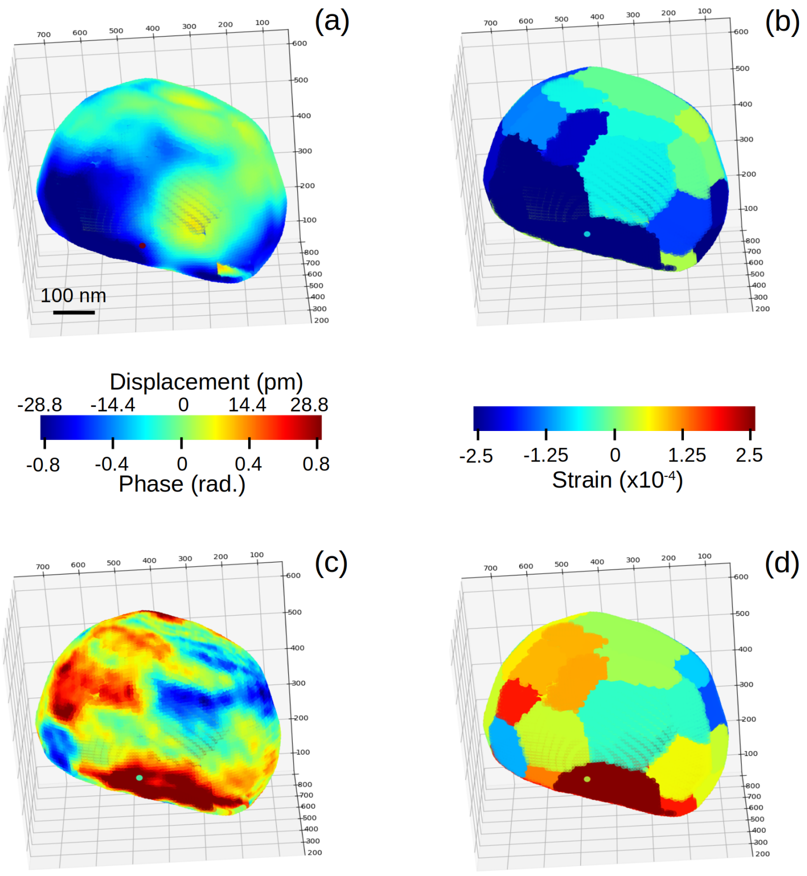

FIG. S15: **Averaged surface displacement and strain per facet calculated using the paraview facet analyser plugin for NP650 during the first scan in Ar atmosphere (S787)**. (a-b) Local and average displacement fields and (c-d) local and average strain fields.

S18. AVERAGE STRAIN VARIATION PER FACET $\Delta\langle\epsilon_{111}\rangle$ AS A FUNCTION OF THEIR FACET TYPE IN THE TWO NPS.

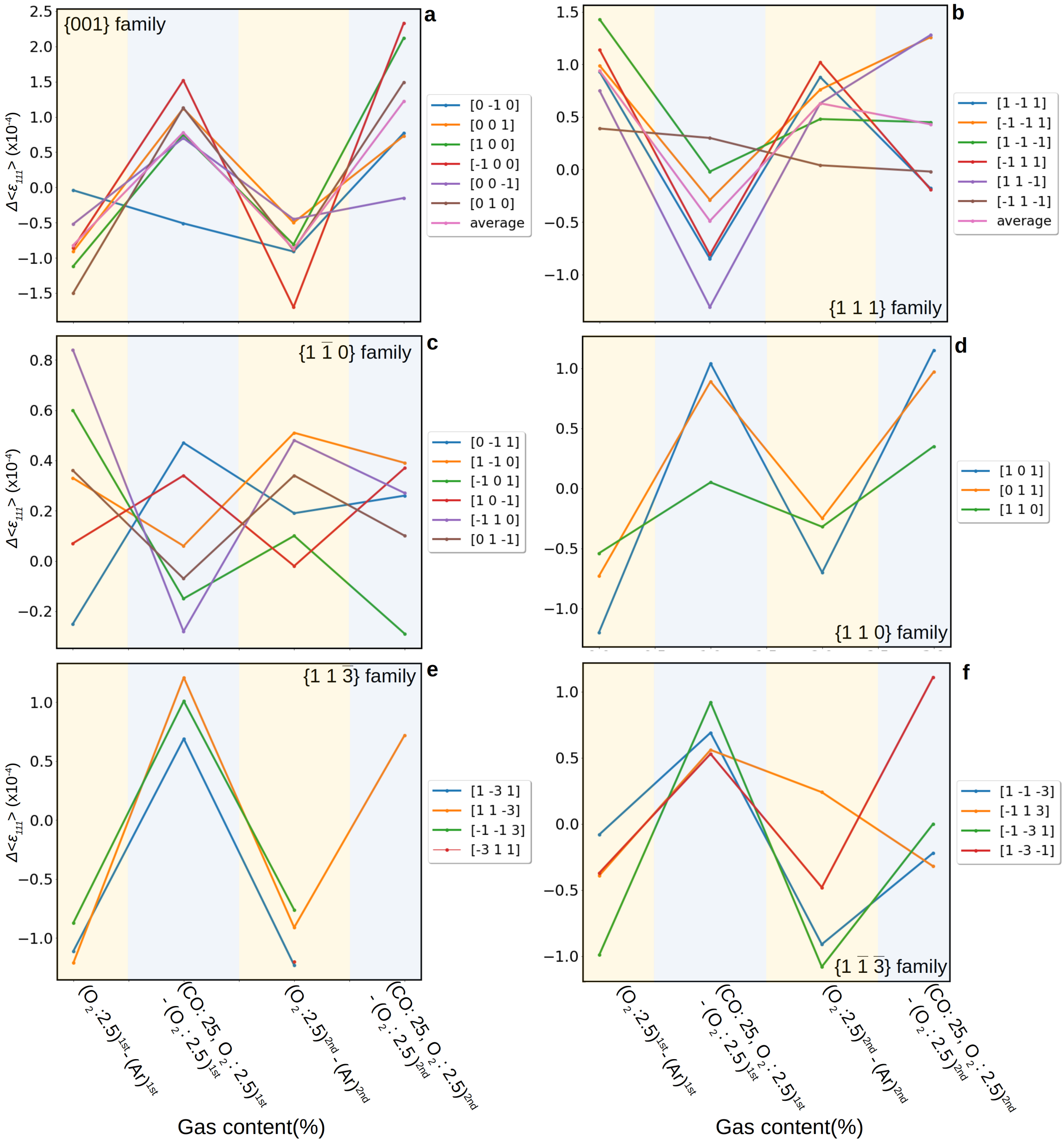

FIG. S16: **Average strain variations per facet as a function of their type (reducing CO oxidation conditions, NP300)**. For a given gas condition, the strain variation, $\Delta\langle\epsilon_{111}\rangle$, is calculated with respect to the previous gas condition for the **a** {001}, **b** {111}, **c** $\{1\bar{1}0\}$, **d** {110}, **e** $\{11\bar{3}\}$ and **f** $\{1\bar{1}\bar{3}\}$ type facets. A yellow background indicates a switch from pure Ar to Ar + O_2 while a blue background indicates a switch from Ar + O_2 to CO oxidation reaction conditions.

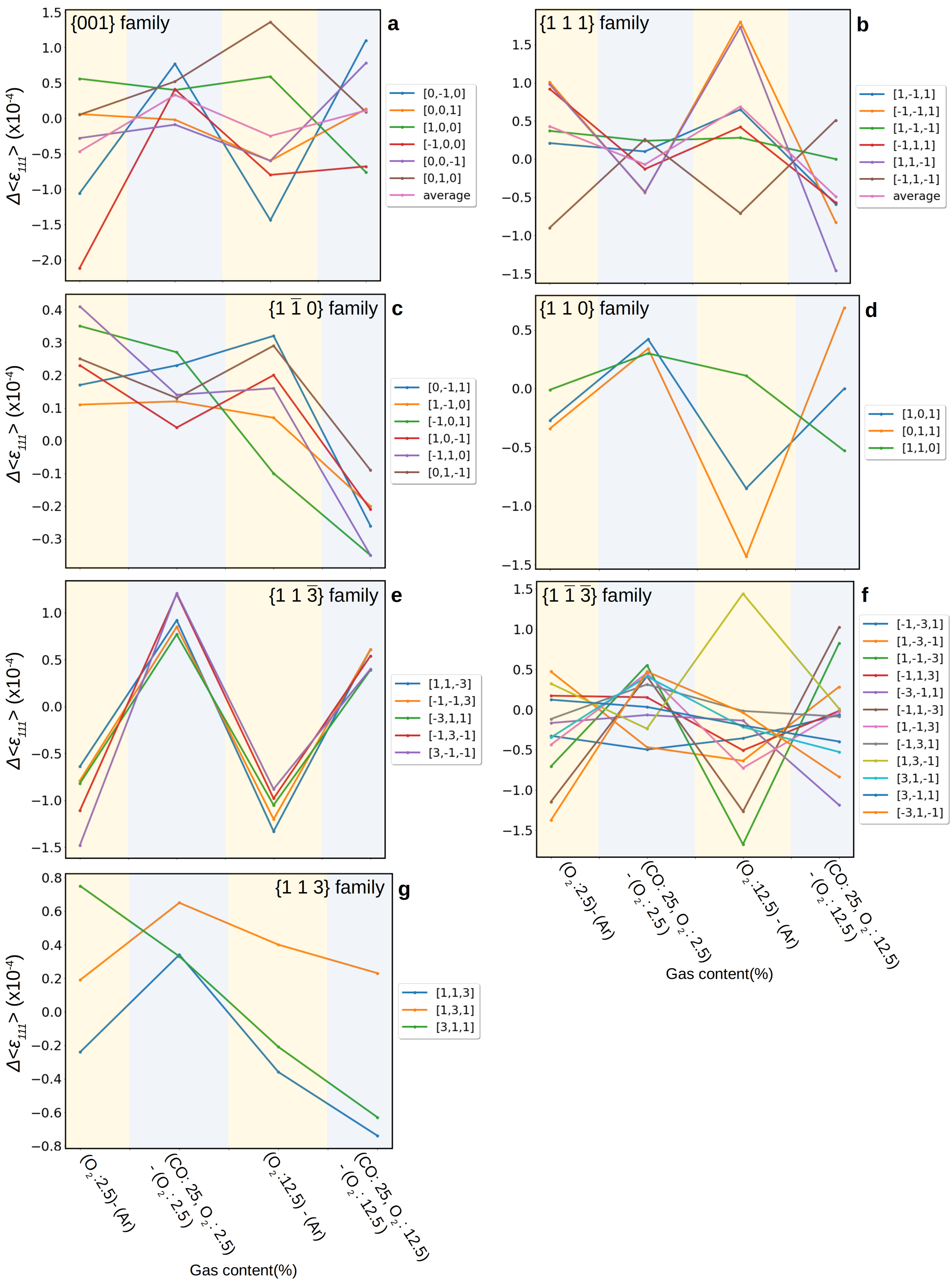

FIG. S17: **Average strain variations in NP650 during the reducing and stoichiometric cycles**. For a given gas condition, the strain variation, $\Delta\langle\epsilon_{111}\rangle$, is calculated with respect to the previous gas condition for the **a** {001}, **b** {111}, **c** $\{1\bar{1}0\}$, **d** {110}, **e** $\{11\bar{3}\}$, **f** $\{1\bar{1}\bar{3}\}$ and **g** {113} type facets. A yellow background indicates a switch from pure Ar to Ar + O_2 while a blue background indicates a switch from Ar + O_2 to CO oxidation reaction conditions.

	Facet type	Ar ϵ_{111} ($\times$ 10^{-4})	Ar + O_2 (2.5%) ϵ_{111} ($\times$ 10^{-4})	Ar + CO (25%) + O_2 (2.5%) ϵ_{111} ($\times$ 10^{-4})
1st cycle	1 0 0	0.78	-0.01	0.77
	1 $\bar{1}$ 1	-0.75	0.11	-0.38
	1 1 $\bar{3}$	1.78	0.95	1.74
	1 $\bar{1}$ $\bar{3}$	0.96	0.89	1.18
	1 $\bar{1}$ 0	0.14	0.46	0.53
	1 1 0	0.56	-0.27	0.39
	1 1 1	0.76	0.24	0.49
	$\bar{1}$ $\bar{1}$ $\bar{1}$	0.54	0.89	1.17
2nd cycle	1 0 0	0.76	-0.12	1.1
	1 $\bar{1}$ 1	-0.68	-0.05	0.38
	1 1 $\bar{3}$	1.69	0.67	1.97
	1 $\bar{1}$ $\bar{3}$	1.03	0.56	0.7
	1 $\bar{1}$ 0	0.2	0.47	0.65
	1 1 0	0.47	0.05	0.87
	1 1 1	0.69	0.34	0.05
	$\bar{1}$ $\bar{1}$ $\bar{1}$	1.21	0.82	1.18

TABLE S6: Average strain per facet subfamilies during the reducing CO oxidation cycles in NP300.

	Facet type	Ar ϵ_{111} ($\times$ 10^{-4})	Ar + O_2 (2.5%) ϵ_{111} ($\times$ 10^{-4})	Ar + CO (25%) + O_2 (2.5%) ϵ_{111} ($\times$ 10^{-4})
1st cycle	1 0 0	0.68	0.21	0.53
(χ = 2)	1 $\bar{1}$ 1	-0.54	-0.11	-0.38
	1 1 $\bar{3}$	1.23	0.26	1.25
	1 $\bar{1}$ $\bar{3}$	0.48	0.27	0.3
	1 1 3	-0.64	-0.41	0.03
	1 $\bar{1}$ 0	0.24	0.49	0.64
	1 1 0	-0.23	-0.43	-0.08
	1 1 1	-0.1	0.36	-0.07
	$\bar{1}$ $\bar{1}$ $\bar{1}$	1.84	1.31	1.43
2nd cycle	1 0 0	0.65	0.41	0.54
(χ = 10)	1 $\bar{1}$ 1	-0.46	0.24	-0.25
	1 1 $\bar{3}$	1.57	0.49	0.99
	1 $\bar{1}$ $\bar{3}$	0.63	0.35	0.18
	1 1 3	-0.18	-0.24	-0.62
	1 $\bar{1}$ 0	0.52	0.67	0.43
	1 1 0	0.05	-0.68	-0.62
	1 1 1	0.23	0.34	-0.27
	$\bar{1}$ $\bar{1}$ $\bar{1}$	1.92	1.19	1.07

TABLE S7: Average strain per facet family during the reducing and stoichiometric cycles in NP650.

Figures S16 and S17 and Tables S6 and S7 further highlight the cyclic behavior of the strain variation during reducing CO oxidation in both NPs. Given the faceted character of the NPs, the initial surface strain state is anisotropic and strongly correlated to the facet type (Tables S5, S6 and S7). In NP300, out of the six $\{1\bar{1}1\}$ side facets, five are in compression, $\langle\langle\epsilon_{111}\rangle_{1\bar{1}1}\rangle$ = -0.75x10^{-4}, while a tensile surface strain is observed in most {100}, $\{11\bar{3}\}$ and $\{1\bar{1}\bar{3}\}$ facets ($\langle\langle\epsilon_{111}\rangle_{100}\rangle$ = 0.78x10^{-4}, $\langle\langle\epsilon_{111}\rangle_{11\bar{3}}\rangle$ = 1.78x10^{-4} and $\langle\langle\epsilon_{111}\rangle_{1\bar{1}\bar{3}}\rangle$ = 0.96x10^{-4}). For the {1 1 0} type facets, the strain state depends on the orientation of the facet normal with respect to the scattering vector $\mathbf{q}_{111}$. The six $\{1\bar{1}0\}$ facets, where the reconstructed surface strain is purely in-plane (*i. e.* parallel to the facet), are almost strain-free on average, while the 3 {110} facets, where the $\mathbf{q}$ vector probes a mix of out-of-plane and in-plane strains exhibit a slight tensile strain ($\langle\langle\epsilon_{111}\rangle_{100}\rangle$ = 0.56x10^{-4}). A similar initial surface strain state is observed in NP650, highlighting the strong

correlation between facet type and initial strain state, which is very well captured by MS simulations (see Figure 4). The introduction of 2.5% O_2 in the gas mixture induces very distinct and reproducible strain variations in NP300 (see Fig. S16 and Table S6) and NP650 (see Fig. S17 and Table S7). The strain evolution is strongly correlated to the facet type and to the initial strain state. As shown in Fig. S16b and Table S6, a relaxation of the compressive strain is observed in the $\{1\bar{1}1\}$ facets, ($\Delta\langle\langle\epsilon_{111}\rangle_{1\bar{1}1}\rangle$ = 0.93x10^{-4} and 0.63x10^{-4} for the first and second cycle, respectively). On average the $\{1\bar{1}1\}$ facets become strain free in Ar + 2.5% O_2 atmosphere (Table S6). Similarly, the binding of oxygen adsorbates induce a relaxation of the surface tensile strain for the $\{100\}$ facets: $\Delta\langle\langle\epsilon_{111}\rangle_{100}\rangle$ = -0.82x10^{-4} (1stcycle) / -0.88x10^{-4} (2ndcycle), $\{11\bar{3}\}$: $\Delta\langle\langle\epsilon_{111}\rangle_{11\bar{3}}\rangle$ = -0.83x10^{-4} (1stcycle) / –1.02x10^{-4} (2nd cycle) , $\{1\bar{1}\bar{3}\}$ facets: $\Delta\langle\langle\epsilon_{111}\rangle_{1\bar{1}\bar{3}}\rangle$ = -0.06x10^{-4} (1stcycle) / –0.47x10^{-4} (2ndcycle) and $\{110\}$ facet families: $\Delta\langle\langle\epsilon_{111}\rangle_{110}\rangle$ = -0.83.1x10^{-4} (1stcycle) / –0.42x10^{-4} (2nd cycle). Overall the strain evolution is very similar in NP650, where the oxygen adsorption also induces a strain relaxation for most facet families (Table S7). In contrast, the opposite trend is observed during CO oxidation in CO excess (χ = 10), where most of the facets come back to their initial strain state. An increase of the tensile strain is therefore observed for the $\{100\}$, $\{11\bar{3}\}$, $\{1\bar{1}\bar{3}\}$ and $\{110\}$ families, while the $\{1\bar{1}1\}$ facets resume their initial compressive strain state during the 1st but not during the 2nd cycle, where a slight tensile strain builds up. Overall, an increase of the magnitude of the surface strain either tensile or compressive is observed for all but one facet families during reducing CO oxidation. This cyclic behavior consisting of a strain relaxation during O_2 exposure and a strain increase during non stoichiometric CO oxidation is well illustrated in Fig. S16. Although less pronounced than in NP300, it is also observed in NP650, not only in non stoichiometric conditions, but also during the subsequent stoichiometric cycle (see Fig. S17). Moreover, Figure S16 also reveals that facets belonging to the same family tend to have a very consistent behavior. This is particularly striking for the $\{100\}$ (Fig. S16a), $\{1\bar{1}1\}$ (Fig. S16b) and $\{11\bar{3}\}$ (Fig. S16e) facet families. Interestingly, this is not the case for the $\{1\bar{1}0\}$ type facets, which exhibit an heterogeneous behavior during the reducing CO oxidation cycles (Fig. S16b). This also explains why on average the cyclic behavior is not observed for this facet type (Fig. 5a). For NP650, with the exception of the $\{11\bar{3}\}$, where all the facets experience a very similar strain evolution (Fig. S17e), the behavior of individual facets tend to be less homogeneous. This is reflected by the smaller amplitude of the oscillations during the gas cycles, in particular for the $\{100\}$ and the $\{1\bar{1}\bar{3}\}$ families (see Fig. 5b).

S19. DFT CALCULATIONS: STRAIN INDUCED BY OXYGEN ADSORPTION.

Family	Exp. NP300 $\Delta\langle\epsilon_{111}\rangle$ ($\times$ 10^{-4})	Exp. NP650 $\Delta\langle\epsilon_{111}\rangle$ ($\times$ 10^{-4})	DFT (1 ML) $\Delta\langle\epsilon_{111}\rangle$ ($\times$ 10^{-4})	DFT (0.75 ML) $\Delta\langle\epsilon_{111}\rangle$ ($\times$ 10^{-4})	DFT (0.5 ML) $\Delta\langle\epsilon_{111}\rangle$ ($\times$ 10^{-4})	DFT (0.25 ML) $\Delta\langle\epsilon_{111}\rangle$ ($\times$ 10^{-4})	DFT (0.125 ML) $\Delta\langle\epsilon_{111}\rangle$ ($\times$ 10^{-4})
1 1 1	-0.45	0.46	-13.51	/	-5.76	-1.56	3.37
1 $\bar{1}$ 1	0.86	0.43	-1.5	/	-0.64	-0.17	0.37
1 0 0	-0.79	-0.47	-1.46	0.37	7.85(010)/8.29(110)	2.24	1.17

TABLE S8: Average strain variation (ϵ_{111}) per facet during O_2 adsorption as a function of oxygen coverage. The strain variations measured in NP300 and NP650 in Ar + 2.5% O_2 atmosphere are indicated as a reference.

Table S8 shows a comparison between the experimental values of $\Delta\langle\epsilon_{111}\rangle$ for the $\{111\}$, $\{1\bar{1}1\}$ and $\{100\}$ facets. To allow a quantitative comparison, the atomic resolved strain values were averaged over the experimental voxel size (6.7 nm). Since the atomic relaxation is mostly perpendicular to the facets, the α angle between the facet normal and the scattering vector $\mathbf{q}_{111}$ has to be taken into account for the calculation of the theoretical average strain in the side $\{100\}$ and $\{1\bar{1}\ 1\}$ facets: $\Delta\langle\epsilon_{111}\rangle^{c}_{100} = \Delta\langle\epsilon_{111}\rangle_{100}\times\cos^2\alpha$. Note that as illustrated in Fig. 4, the strain field strongly depends on the NP shape and boundary conditions, which results in large atomic displacements not only perpendicular to the facets but also parallel to the facets (for instance for the $\{11\bar{3}\}$ facets). These displacements can not be captured by the DFT calculations, which could contribute to explain the discrepancy between the experimental values and the DFT results. The results displayed in Table S8 suggest a low coverage on the $\{111\}$ and $\{1\bar{1}1\}$ facets (between 0.125 and 0.25 ML) during oxygen exposure in Ar + 2.5% O_2 atmosphere. In contrast a high coverage is expected on the $\{100\}$ facet (between 0.75 and 1 ML). The DFT calculations therefore confirm that adsorption occurs preferentially on low coordinated facets that are initially in tension, in good agreement with the d-band model.

S20. AVERAGE STRAIN VARIATION PER FACET $\Delta\langle\epsilon_{111}\rangle$ AS A FUNCTION OF THEIR FACET TYPE OR LOCATION (STOICHIOMETRIC CONDITIONS, NP300).

	Facet type	CO (25%) + O_2 (2.5%) ϵ_{111} ($\times 10^{-4}$)	O_2 (12.5%) ϵ_{111} ($\times 10^{-4}$)	CO (25%) + O_2 (12.5%) ϵ_{111} ($\times 10^{-4}$)	O_2 (12.5%) ϵ_{111} ($\times 10^{-4}$)
1st cycle	1 0 0	1.1	0.38	-0.29	0.52
	$1\ \bar{1}\ 1$	0.38	0.42	-0.18	0.11
	$1\ 1\ \bar{3}$	1.97	1.11	0.72	2.01
	$1\ \bar{1}\ \bar{3}$	0.7	-0.09	-0.83	1.13
	$1\ \bar{1}\ 0$	0.65	0.79	0.4	0.43
	1 1 0	0.87	1.36	0.72	0.35
	1 1 1	0.05	0.17	0.7	0.44
	$\bar{1}\ \bar{1}\ \bar{1}$	1.18	1.56	1.02	1.81

TABLE S9: Average strain per facet family during the stoichiometric cycle in NP300.

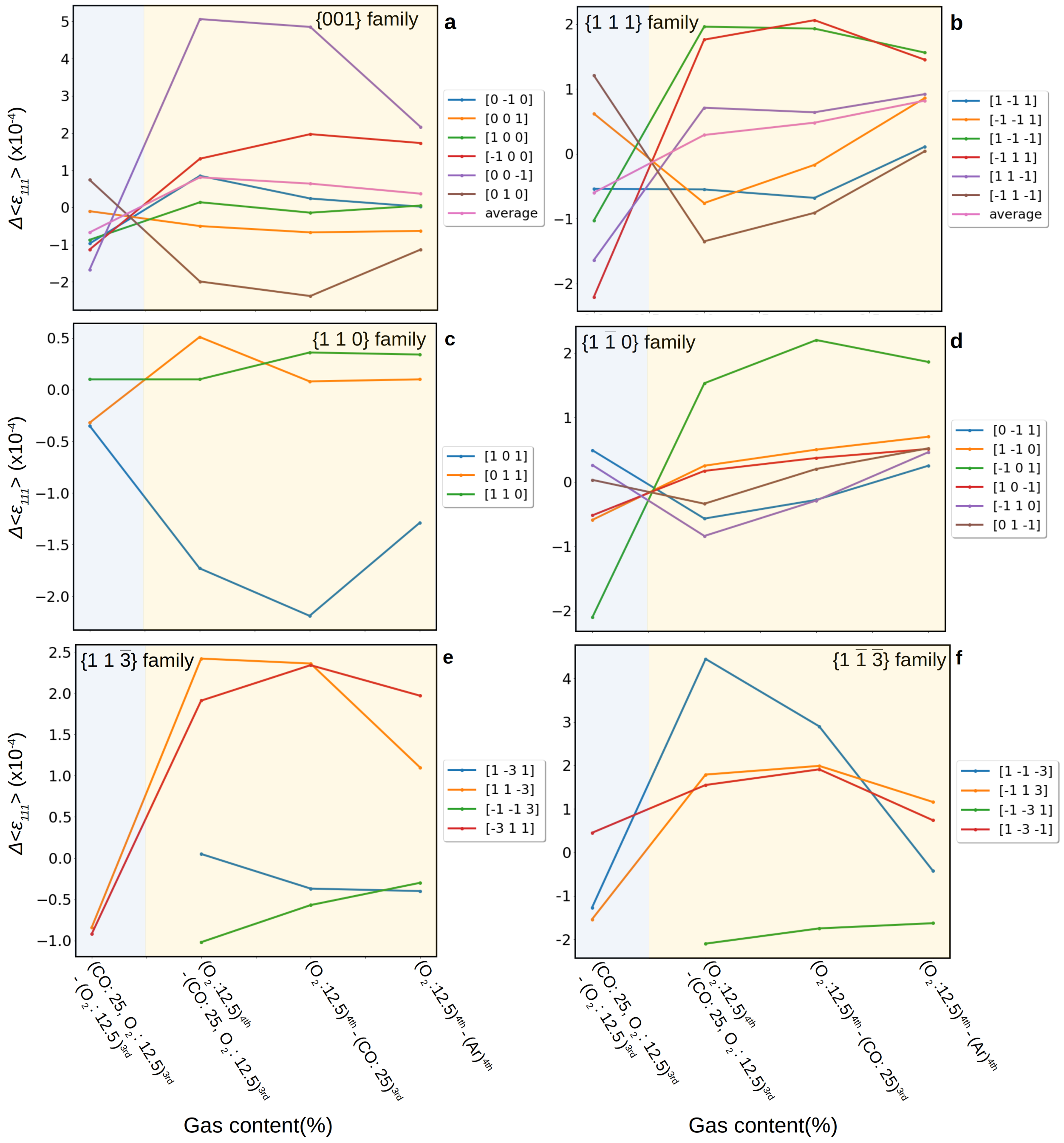

FIG. S18: **Average strain variations per facet as a function of their type (stoichiometric conditions, NP300).** The label on the x-axis shows the reference gas condition used to calculate the strain variation for each data point. For a given gas condition, the strain variation, $\Delta\langle\epsilon_{111}\rangle$, is calculated with respect to the previous gas condition for the **a** {001}, **b** {111}, **c** {110}, **d** $\{1\bar{1}0\}$, **e** $\{11\bar{3}\}$ and **f** $\{1\bar{1}\bar{3}\}$ type facets. The blue background indicates a switch from Ar + O_2 atmosphere to CO oxidation reaction conditions while the yellow background indicates a switch from either CO oxidation reaction conditions or Ar + CO or pure Ar atmosphere to Ar + O_2.

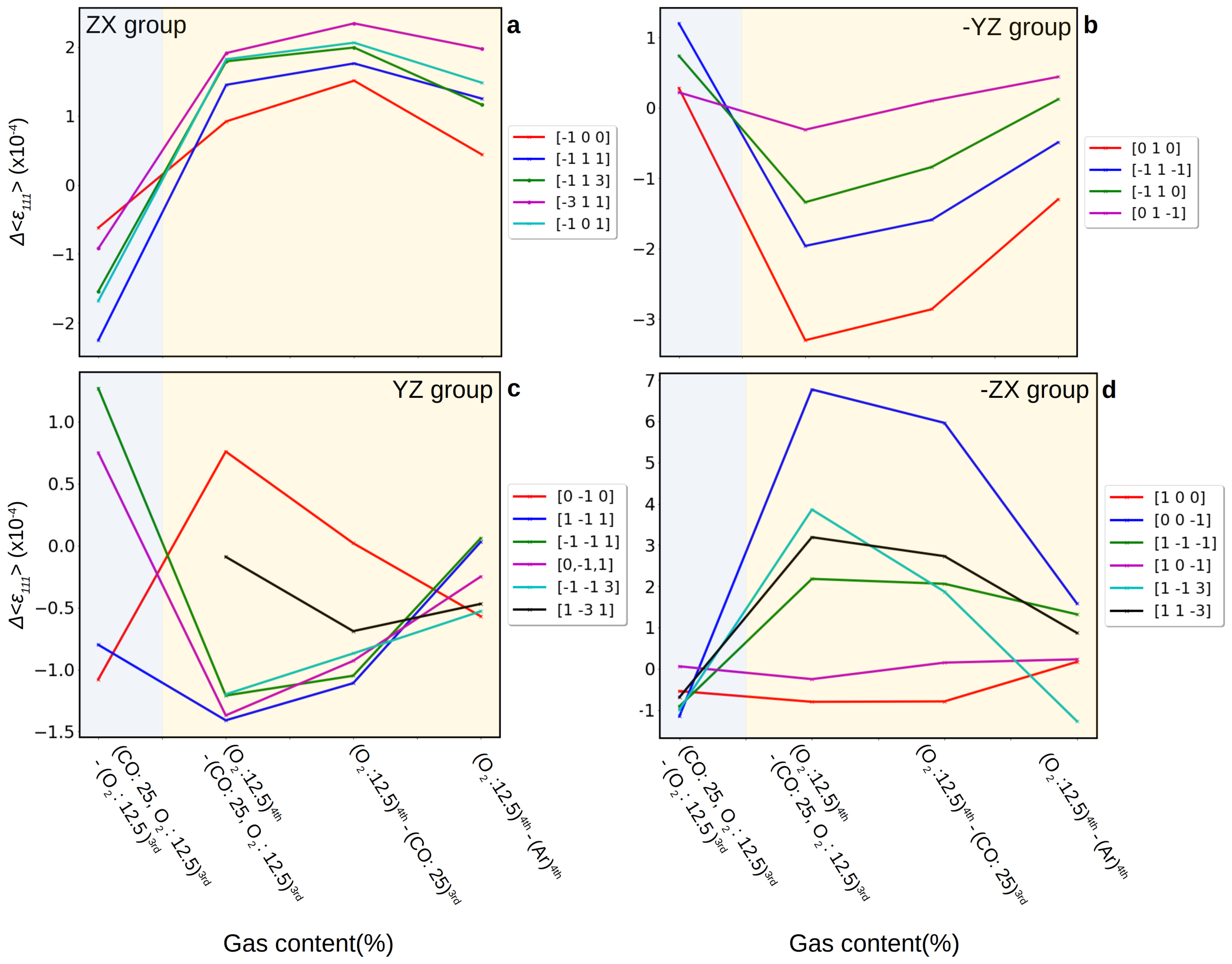

FIG. S19: **Average strain variations per facet as a function of their location (stoichiometric conditions, NP300)**. For a given gas condition, the strain variation, $\Delta\langle\epsilon_{111}\rangle$, is calculated with respect to the previous gas condition for facets localised in the **a** ZX, **b** -YZ, **c** YZ and **d** -ZX regions. The label on the x-axis shows the reference gas condition used to calculate the strain variation for each data point. The blue background indicates a switch from Ar + O_2 atmosphere to CO oxidation reaction conditions while the yellow background indicates a switch from either CO oxidation reaction conditions or Ar + CO or pure Ar atmosphere to Ar + O_2.

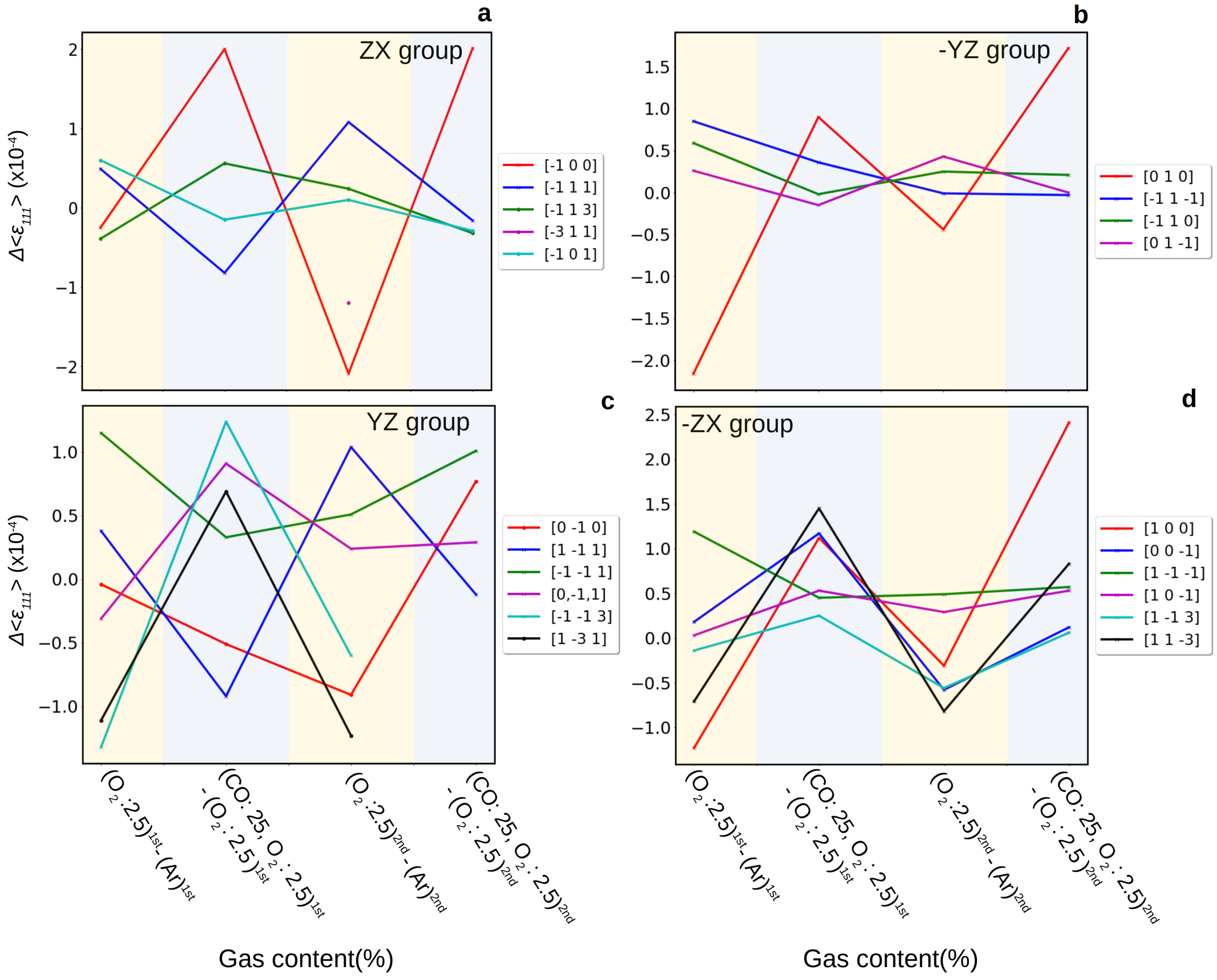

FIG. S20: **Average strain variations per facet as a function of their location (reducing CO oxidation conditions, NP300)**. For a given gas condition, the strain variation, $\Delta\langle\epsilon_{111}\rangle$, is calculated with respect to the previous gas condition for facets localised in the **a** ZX, **b** -YZ, **c** YZ and **d** -ZX regions. A yellow background indicates a switch from pure Ar to Ar + O_2 while a blue background indicates a switch from Ar + O_2 to CO oxidation reaction conditions.

If the facet dependent character is mostly retained during stoichiometric CO oxidation in NP650, this is not the case for NP300. This is illustrated in Fig. S18 showing that individual facets belonging to the same family do not follow the same trend during stoichiometric CO oxidation. For instance, two of the facets belonging to the $\{1\bar{1}1\}$ family experience a tensile strain (the $[\bar{1}\bar{1}1]$ and $[\bar{1}1\bar{1}]$), while the remaining four go into compression (see Fig. S18a). Similar observations can be drawn for the remaining families. At odds with previous works on single crystal surfaces and with the trends observed in reducing CO oxidation conditions, the analysis of the average strain evolution reveals that the strain evolution is weakly correlated to the facet type during stoichiometric CO oxidation: facets can go into tension or compression independently on their crystallographic orientation. In contrast, facets localised in the same region of the nanocrystal tend to exhibit a more similar behavior (Fig. S19). This is particularly true for the facets localised in the regions labeled ZX and -YZ, which go into compression (Fig. S19a) and tension (Fig. S19b), respectively. For the sake of comparison, the strain evolution in the four regions (ZX, -ZX, YZ and -YZ) is shown during the reducing CO oxidation cycles (Fig. S20). In these conditions, the strain variations are strongly correlated to the facet type, and therefore facets belonging to the different regions, with different surface termination exhibit a very heterogeneous behavior. This further highlights the existence of two regimes in NP300. A facet dependent regime in reducing CO oxidation conditions, where the strain evolution depends on the facet type and a localised regime in stoichiometric conditions, where the strain evolution is predominantly controlled by the facet position in the NP. The strain variations remain mostly independent of the facet type during the subsequent O_2 exposure (Ar + 12.5%

O_2, 4th cycle). As illustrated in Fig. S18, facets that experienced compressive (tensile) strain during stoichiometric CO oxidation go into tension (compression) in O_2 atmosphere, independently of their type. On the other hand, the localised character of the strain variations is retained during O_2 exposure: the ZX and -ZX regions that experienced a compressive strain during CO oxidation go into tension in O_2 atmosphere, while the opposite trend is observed for the neighboring YZ and -YZ regions. This allows to relax most of the large surface strain that was induced by the stoichiometric oxidation. Interestingly and as illustrated in Figs. S18 and S19, this surface strain relaxation is initiated before the O_2 exposure, as illustrated by the overall decrease of $\Delta\langle\epsilon_{111}\rangle$ during the Ar + 25% CO and Ar exposures preceding the O_2 exposure. This overall decrease of the average strain per facet is consistent with the decrease of both the microstrain (Fig. 3a) and strain field energy (Fig. 3c), which starts rapidly after the end of the stoichiometric CO oxidation. This relaxation is also accompanied by a decrease of the extent of the compressive regions at the NP / substrate interface (Fig. S8h-j), suggesting that a reorganisation of the interfacial dislocation network can also contribute to accommodate the large strain induced during the gas reaction.

S21. AVERAGE LATTICE DISPLACEMENT AND STRAIN FOR ALL FACETS FOR NP300.

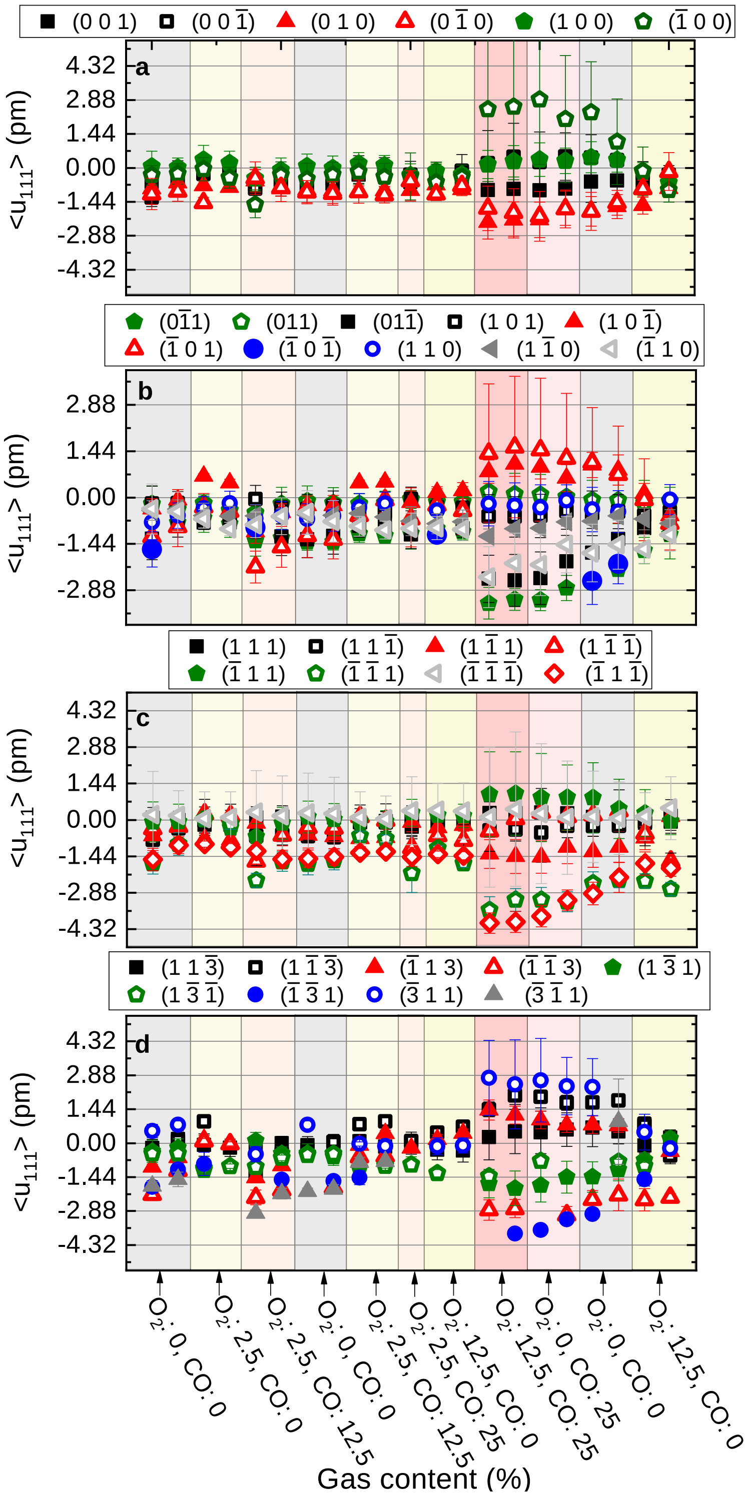

FIG. S21: **Evolution of the average lattice displacement along the [111] direction for all the facets of the four different families of facets for NP300**. **a** {100}, **b** {110}, **c** {111} and **d** {113} facets. The error bars represent the standard deviation of the displacement per facet. The large inhomogeneous displacement observed during the stoichiometric CO oxidation reaction is reflected by large error bars.

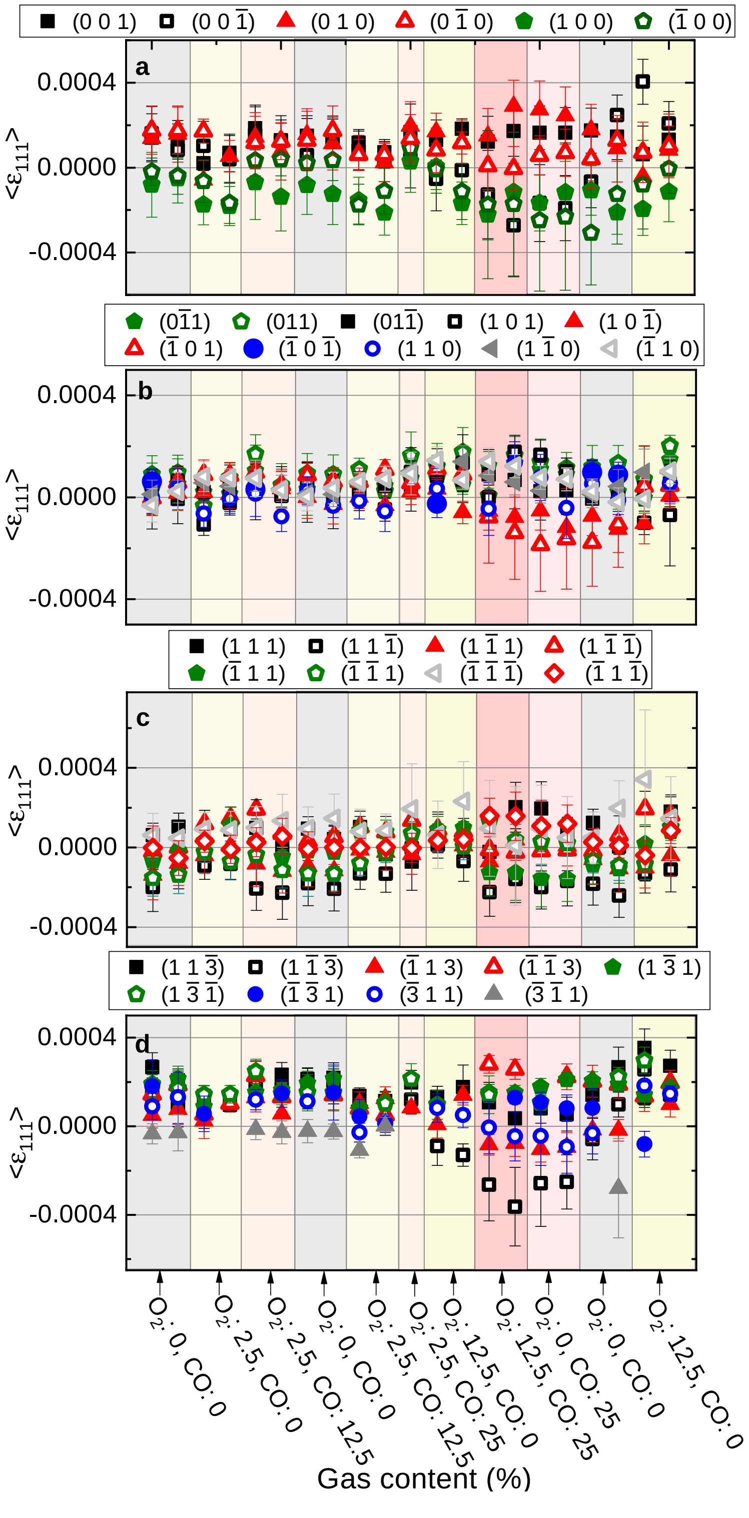

FIG. S22: **Evolution of the average strain along the [111] direction for all the facets of the four different families of facets for NP300**. **a** {100}, **b** {110}, **c** {111} and **d** {113} facets. The error bars represent the standard deviation of the strain per facet. The large inhomogeneous displacement observed during the stoichiometric CO oxidation reaction is reflected by large error bars.

S22. EVOLUTION OF THE AVERAGE DISPLACEMENT FIELD PER FACET $\Delta\langle U_{111}\rangle_{facet}$ AT THE TOP AND BOTTOM OF THE NANOPARTICLE.

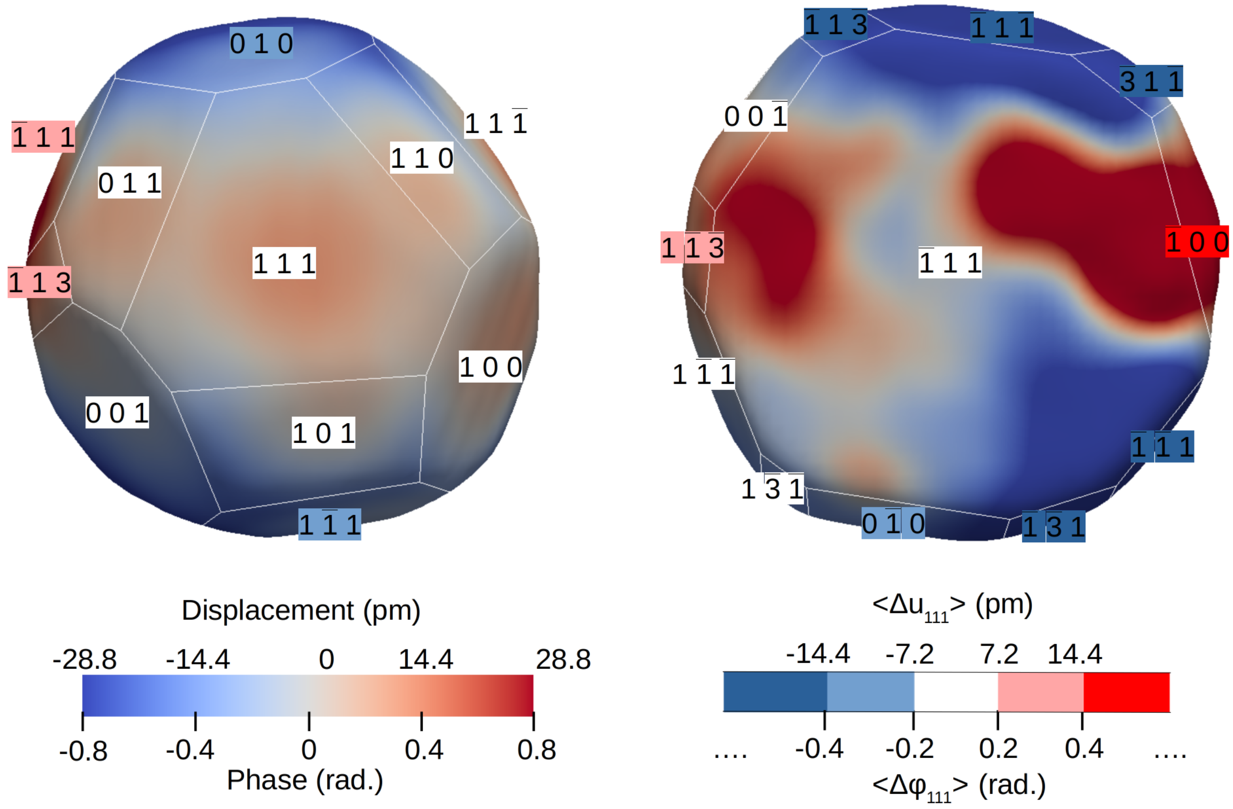

FIG. S23: **Phase variation during CO oxidation**. Comparison between top (left view) and bottom (right view) surfaces for NP300.

Family	$\Delta\langle u_{111}\rangle$ ($\times$ 10^{-4}) (top)	$\Delta\langle u_{111}\rangle$ ($\times$ 10^{-4}) (bottom)
1 0 0	0.19	0.38
1 $\bar{1}$ 1	0.22	0.45
1 1 0	0.05	0.38
1 1 3	0.27	0.34
Average	0.18	0.39

TABLE S10: Average strain variation per facet family for facets located at the top and bottom of NP300.

Figure S23 and Table S10 show the variation of the surface lattice displacement for facets located at the top and bottom of NP300 during stoichiometric CO oxidation. The displacement variations for the facets located at the bottom of the NP are significantly larger than the variations on the top facets. Large strain/displacement variations are characteristic of high activity regimes as suggested by Plodinec *et al.* [6], which would suggest that preferential sites for CO_2 formation are predominantly located on the facets located close to the interface. This indicates that the thermoelastic strain induced during the cooling of the NP at the substrate/NP interface can affect the reactivity and confirms the importance of metal-support interaction to improve performance in catalysis.

S23. MASS SPECTROMETRY.

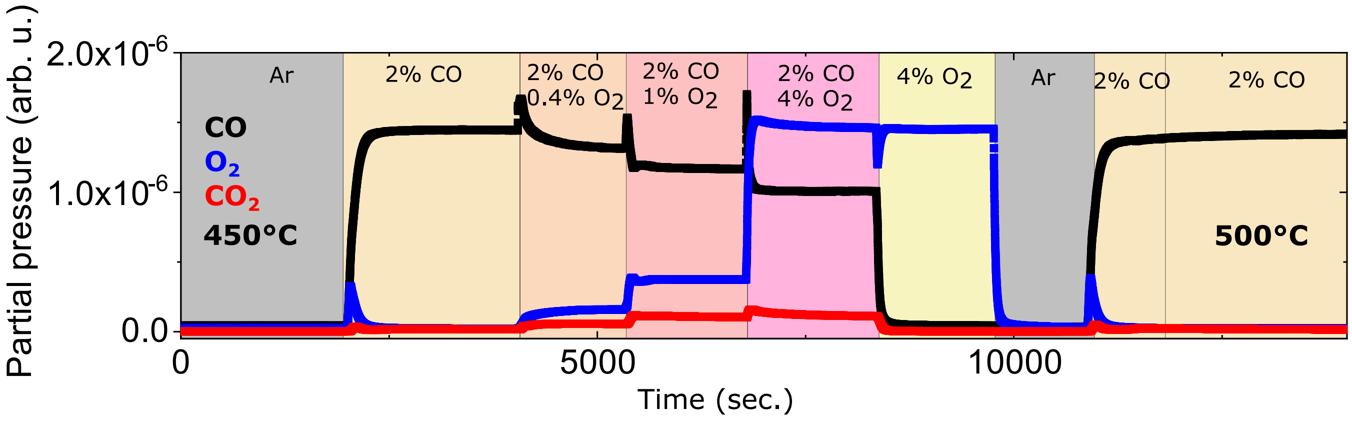

FIG. S24: **Mass spectrometry from a different sample with Pt particles very similar in size to the sample measured in the manuscript**. Formation of CO_2 during CO oxidation at 450°C.

Figure S24 displays the CO conversion into CO_2 as a function in reducing, stoechiometric and oxidizing conditions. Note that the measurements were carried out on a different sample with a similar size distribution of Pt NPs.

S24. HISTOGRAM OF THE DISPLACEMENTS U_{111} OF THE SURFACE VOXELS FOR NP300.

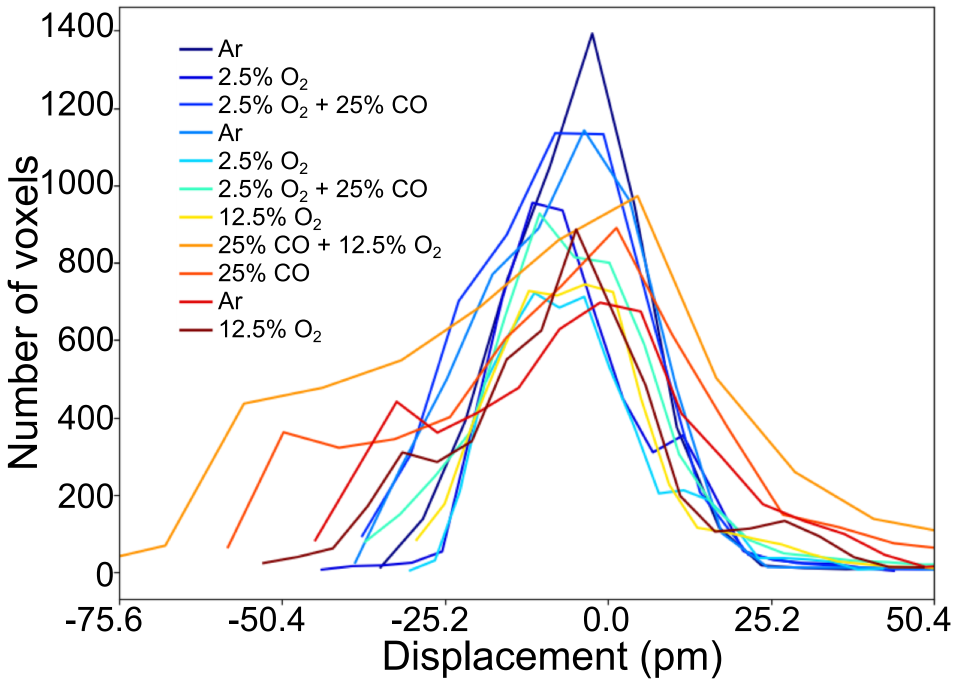

FIG. S25: **Surface voxels.** Histogram of the displacement of the surface voxels as a function of gas mixtures for NP300.

Figure S25 shows the evolution of the surface lattice displacement during the two non stoichiometric cycles and the stoichiometric cycle in NP300. A large and inhomogeneous surface displacement builds up during stoichiometric CO oxidation, in good agreement with the increase of the microstrain (Fig. 2a) and strain-field energy (Fig. 2c). The strain relaxation is initiated during the subsequent 25% CO and Ar exposure, most likely through a reorganisation of the interfacial dislocation network. With the introduction of 12.5% O_2, the FWHM of the histogram almost completely comes back to is initial value, suggesting that these oxidative conditions allow to oxidize the remaining CO adsorbates on the surface step sites.

Supplementary References

[1] Chapman, H. N. *et al.* High-resolution *Ab Initio* three-dimensional x-ray diffraction microscopy. *J. Opt. Soc. Am. A* **23**, 1179–1200 (2006).

[2] Cherukara, M. J., Cha, W. & Harder, R. J. Anisotropic nano-scale resolution in 3D bragg coherent diffraction imaging. *Appl. Phys. Lett.* **113**, 203101 (2018).

[3] Carnis, J. *et al.* Towards a quantitative determination of strain in Bragg Coherent X-ray Diffraction Imaging: Artefacts and sign convention in reconstructions. *Sci. Rep.* **9**, 1–13 (2019).

[4] Robinson, I. & Harder, R. Coherent X-ray diffraction imaging of strain at the nanoscale. *Nat. Mater.* **8**, 291–298 (2009).

[5] Clark, J. N. *et al.* Ultrafast three-dimensional imaging of lattice dynamics in individual gold nanocrystals. *Science* **341**, 56–59 (2013).

[6] Plodinec, M., Nerl, H. C., Girgsdies, F., Schlögl, R. & Lunkenbein, T. Insights into Chemical Dynamics and Their Impact on the Reactivity of Pt Nanoparticles during CO Oxidation by Operando TEM. *ACS Catal.* **10**, 3183–3193 (2020).
